# Effectiveness of GLP-1 RAs and SGLT2 inhibitors in preventing T2DM in high-risk patients: an updated systematic review and meta-analysis

**DOI:** 10.3389/fcdhc.2025.1694808

**Published:** 2025-12-10

**Authors:** Georgios I. Tsironikos, Vasiliki Tsolaki, George E. Zakynthinos, Despoina Kyprianidou, Vasiliki Rammou, Thomas Antonogiannis, Theodoros Mprotsis, Epameinondas Zakynthinos, Alexandra Bargiota

**Affiliations:** 1 Department of Research for General Medicine and Primary Health Care, Faculty of Medicine, University of Ioannina, Ioannina, Greece; 2 Department of Critical Care, University Hospital of Larissa, Faculty of Medicine, University of Thessaly, Larissa, Greece; 3 3rd Department of Cardiology, “Sotiria” Chest Diseases Hospital, Medical School, National and Kapodistrian University of Athens, Athens, Greece; 4 Medical School, National and Kapodistrian University of Athens, Athens, Greece; 5 Faculty of Medicine, University of Thessaly, Larissa, Greece; 6 Department of Biomathematics, University of Thessaly, Faculty of Medicine, Thessaly, Greece; 7 Department of Internal Medicine-Endocrinology, University Hospital of Larissa, Faculty of Medicine, University of Thessaly, Larissa, Greece

**Keywords:** diabetes, diet, exercise, lifestyle, nutrition, GLP-1 RAs, physical activity, SGLT2 inhibitors

## Abstract

**Introduction:**

There are conflicting results and limited data regarding the individual effectiveness of glucagon-like peptide 1 receptor agonists (GLP-1 RAs) and sodium-glucose cotransporter 2 (SGLT2) inhibitors and their combined action in preventing type 2 diabetes mellitus (T2DM) in high-risk adults. An updated investigation is warranted. We aimed to explore their effectiveness in preventing T2DM in high-risk patients and assess changes in body weight/body mass index (BMI), glycemic parameters, and safety.

**Materials and methods:**

PubMed, Cochrane Library Central Register of Controlled Trials, and Scopus were searched for eligible randomized controlled trials (RCTs), and a systematic review (SR) and meta-analysis (MA) were conducted. GRADE assessment was conducted for rating the overall certainty of evidence.

**Results:**

All 24,157 participants in 10 GLP-1 RA RCTs were overweight/obese. Compared to placebo, GLP-1 RAs reduced T2DM incidence (OR 0.51; 95% CI 0.28, 0.94; *P*-value 0.03), and 2.4 mg of semaglutide was overall effective (OR 0.38; 95% CI 0.16, 0.94; *P*-value < 0.0001). Subgroup analysis indicated effectiveness in patients more than 50 years across the world, in cardiovascular disease, after 100 weeks, and during the post-intervention period. Liraglutide was not overall effective. However, subgroup analyses demonstrated effectiveness for studies that were performed worldwide, for women more than 40 years, at 3.0 mg daily, after 55 weeks of administration, and only during intervention. Exenatide was not effective. Heterogeneity was large (Q 54.56, *P*-value < 0.0001; *I*² 84%, 95% CI 74%, 89%), and MA was performed using the random-effects model. Heterogeneity was explained by countries’ performance in semaglutide- and liraglutide-based RCTs and participants’ mean age, dosage, duration, and post-intervention evaluation in liraglutide-based RCTs. Sensitivity analyses considering studies with post-intervention assessment and studies with the largest sample size and dropout rate more than 5% in semaglutide-based RCTs explain further heterogeneity. The quality of evidence was low. Compared to placebo, GLP-1 RAs reduced weight (kg) and BMI (kg/m²) (mean difference –6.35, *P*-value < 0.00001 and –2.46, *P*-value < 0.00001, respectively). Finally, GLP-1 RAs were safe (OR for adverse events 1.01; *P*-value 0.95).

**Conclusions:**

GLP-1 RAs may prevent diabetes in high-risk adults and ameliorate body and glycemic factors. Their effectiveness should be considered carefully due to the low quality of evidence. No safety issues were identified. Future investigation is necessary to provide consistency of estimations.

**Systematic Review Registration:**

OSF Registration, identifier DOI 10.17605/OSF.IO/8XH4

## 1 Introduction

Obesity and type 2 diabetes mellitus (T2DM), often termed “diabesity,” are currently considered a pandemic (1, 2). The prevalence of T2DM is increasing worldwide, with a faster rate of incidence in developed countries (3, 4). Overweight or obesity consists of a T2DM risk factor (5). Additional risk factors include family history of DM in first-degree relatives, history of gestational DM (GDM) or polycystic ovary syndrome (PCOS), prediabetes, hypertension (HY) or antihypertensive treatment, hypertriglyceridemia (>250 mg/dL), cardiovascular disease (CVD), and non-alcoholic fatty liver (NAFLD) (5, 6).

Glucagon-like peptide 1 receptor agonists (GLP-1 RAs), the natural homologs of the incretin hormone GLP-1, have been approved for weight management (7); 2.4 milligrams (mg) of semaglutide once weekly or 3.0 mg of liraglutide once daily is effective in treating obesity (7). Additionally, several systematic reviews (SRs) and meta-analyses (MAs) have demonstrated that sodium-glucose cotransporter 2 (SGLT2) inhibitors lead to significant weight loss in overweight or obese adults (8–10).

Despite GLP-1 RAs’ significant anti-obesity effects, SRs and MAs show conflicting results on their effectiveness in preventing T2DM (11–13). Hemmingsen et al. in 2017 demonstrated no effect of GLP-1 RAs in preventing T2DM in high-risk individuals (11). However, two other SRs and MAs evaluating patients with prediabetes and obesity reported a positive impact (12, 13). In the present study, we will review, synthesize, and present all available and most recent data concerning the effectiveness of GLP-1 RAs in preventing T2DM in high-risk adults. Additionally, we will assess the effect of GLP-1 RAs regarding specific clinical areas: 1) effectiveness of any drug, 2) optimal dosage, 3) effective duration, 4) post-intervention effectiveness, and 5) patients with benefits. We will also evaluate the effectiveness of SGLT2 inhibitors alone or combined with GLP-1 RAs in high-risk adults. Finally, we will assess changes in body weight, body mass index (BMI), hemoglobin A1c (HbA1c), fasting plasma glucose (FPG), 2-hour (h) oral glucose tolerance test (OGTT), and safety.

## 2 Materials and methods

This study was pre-registered in the Open Science Framework (OSF) (Registration DOI 10.17605/OSF.IO/8XH4G). The SR was performed according to the Preferred Reporting Items for SR and MA (PRISMA) extension guideline for complex interventions (14).

### 2.1 Search strategy and eligibility criteria

We searched PubMed, Cochrane Library Central Register of Controlled Trials (CENTRAL), and Scopus for eligible RCTs (from 01/01/2000 to 31/07/2024). According to our protocol, we performed a wide search for pharmaceutical and lifestyle interventions aiming at preventing diabetes in high-risk persons. The keywords were related to diabetes mellitus; drugs including GLP-1 RAs, SGLT2 inhibitors, and metformin; lifestyle; diet or nutrition; and exercise or physical activity (PA) (**Supplementary Table S1**). The Cochrane collaboration search algorithm for RCTs was applied in PubMed, and the same keywords were used in CENTRAL and Scopus (**Supplementary Table S1**).

Duplicates were removed using EndNote 21.1. Four investigators (GIT, VT, GEZ, DK) screened the databases. Potentially eligible RCTs were retrieved in full text and checked based on title and/or abstract. A fifth contributor (AB) checked on the studies that the four investigators (GIT, VT, GEZ, DK) could not decide on, and discrepancies were resolved through consensus.

The inclusion of studies was based on the PICO (population, intervention, control, outcome) approach. Only studies in the English language were included. Trials including adult participants with any diabetes risk factor, using interventions of GLP-1 RAs and/or SGLT2 versus placebo, or pharmaceutical interventions combined with lifestyle interventions versus placebo and the same lifestyle intervention in a comparator arm, with reports of new T2DM cases were included.

For the diagnosis of diabetes, we accepted any appropriate diagnostic method. We accepted the final reported incidence for studies that reported the occurrence of T2DM in different time periods.

We excluded pilot or feasibility RCTs, conference proceedings, RCTs reporting subgroup or *post hoc* analyses, or those not considering diabetes outcome.

### 2.2 Data extraction

We extracted the first name of the author, publication year, country, type of RCT with the number of clusters or centers if clustered or multicenter, studies’ duration, and dropout rate. We also extracted follow-up duration for studies assessing post-intervention incidence of diabetes. The total sample size, participants’ characteristics (gender, age, ethnicity, risk factors for T2DM, baseline HbA1c), and interventions’ characteristics (dosage, duration) were recorded.

For our primary outcome, we extracted diabetes assessment as a primary or secondary outcome, the number of high-risk patients that were analyzed for T2DM, the events of T2DM, and the diagnostic modalities. For our secondary outcomes, we extracted mean differences with standard deviations of body weight, BMI, HbA1c, FPG and 2-h OGTT changes, and potential adverse events.

The data extraction was performed by two researchers (GIT, VT) with the contribution of a third investigator (AB), where the two researchers could not decide.

### 2.3 Quality assessment of the studies and rating of overall evidence

The quality assessment of eligible RCTs was performed using the revised Cochrane Collaboration risk of bias tool 2 (15). The Grading of Recommendations, Assessment, Development, and Evaluation (GRADE) tool (GRADEpro, version 3.6.1 McMaster University, 2011) was used for rating the overall certainty of evidence.

### 2.4 Statistical analysis

We performed an MA to combine the events of T2DM using all available data. The statistically significant level for Cochran’s *Q* statistic was set at *P*-value <0.1 and for other analyses at *P*-value <0.05 (16). The Cochran’s *Q* statistic was used for the assessment of heterogeneity (16), and the *I*² index was used for quantification (<25%, low; 25%–49%, moderate; 50%–75%, large; >75%, very large) (17, 18). Both fixed-effects (FE) and random-effects (RE) models of MA were implemented. If heterogeneity was large, the MA was performed by the RE model (17, 18).

Subgroup analyses were performed based on the characteristics of the studies (similar countries, follow-up duration), populations (gender, mean age, diabetes risk factor), and interventions (dosage, duration). Also, subgroup analyses were performed for studies where T2DM was assessed as a primary or secondary outcome. Sensitivity analyses were conducted to assess the effect of studies with the largest sample size, with follow-up of participants, and with the largest dropout rate.

Publication bias was assessed optically by funnel plot (symmetrical inverted funnel in the absence of bias) and statistically by Egger’s test (19).

We converted the units of reported continuous variables, where needed for harmonization, and MAs were performed by the mean difference effect measure (16). For large heterogeneity, we applied the RE model (17, 18).

The main analyses were performed using the Review Manager software version 5.4.1 (Cochrane Collaboration, London, UK) and Eggers’ test by the Statistical Package for the Social Sciences (SPSS) software version 29.0 (SPSS, Inc., Chicago, IL, USA).

## 3 Results

**Figure 1:**
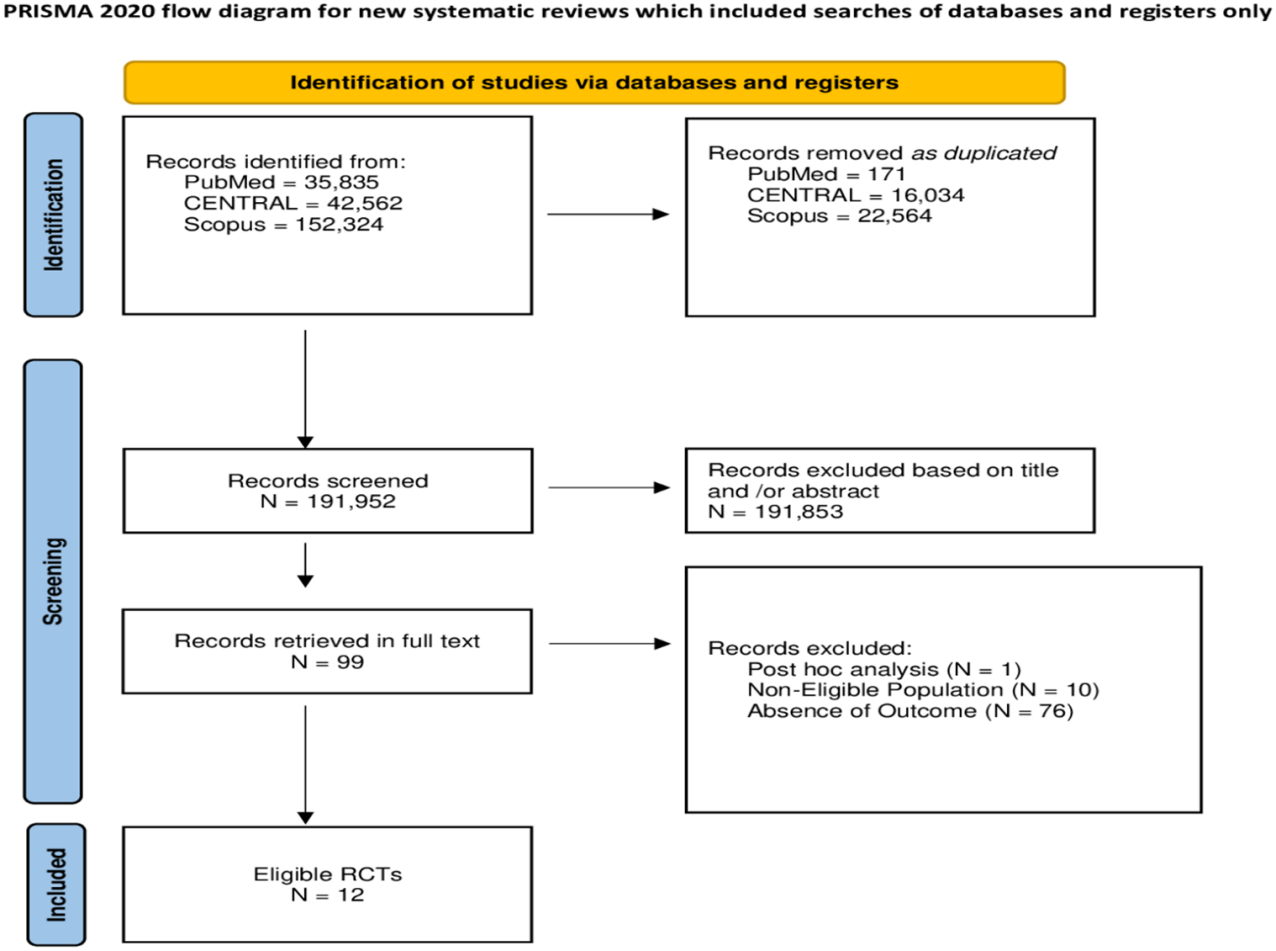
Flowchart of the selection procedure of studies.

The search yielded 230,721 items: 38,679 were duplicates and removed, 191,952 records were considered for potential eligibility, 191,853 items were excluded based on title and/or abstract, and 99 studies were retrieved in full text and checked. Out of 99 potentially eligible studies, 77 were not accepted. Finally, 12 studies were included (**Figure 1**).

Among the 12 eligible trials (20–31), 10 assessed GLP-1 RAs (20, 21, 23–27, 29–31), 5 evaluated semaglutide (25, 26, 29–31), 4 examined liraglutide (21, 23, 24, 27), and 1 assessed exenatide (20). One RCT evaluating SGLT2 inhibitor included dapagliflozin (28), and the RCT assessing both GLP-1 RA and SGLT2 inhibitor included exenatide and dapagliflozin (24).

### 3.1 Characteristics of eligible studies

#### 3.1.1 GLP-1 RA RCTs

The RCTs with GLP-1 RA interventions had a parallel design and were published between 2010 and 2024 (20, 21, 23–27, 29–31). Their duration varied between 28 and 243 weeks, and dropout rates were between <1% and 33% (20, 21, 23–31).

Eight studies were multicenter (21, 23–26, 29–31). The number of centers varied between 2 and 804 (21, 23–26, 29–31). An RCT involving 2 centers was conducted in Denmark (24); RCTs involving 23 centers were conducted in China, South Korea, Hong Kong, and Brazil (29); those with 30 centers in Canada, United Kingdom (UK), Spain, Denmark, and Finland (31); those with 37 centers in the United States of America (USA), Canada, UK, Germany, and Japan (25); those with 41 centers in the USA, Canada, Italy, Spain, Hungary (26); those with 191 centers in 27 countries including all continents (21, 23); and those with 804 centers in 41 countries including also all continents (30). Two RCTs were single-center studies (20, 27), and they were conducted in the USA (20) and Denmark (27) (**Table 1**).

**Table 1:** Characteristics of eligible RCTs.

First author’s name, publication year	Countries	No. of centers	Study duration (wks)	Follow-up duration for T2DM (wks)	Dropout rate (%)
GLP-1 RAs RCTs					
Semaglutide-based RCTs					
Wilding, 2022 (25)	USA, Canada, UK, Germany, Japan	37	120	52	6
Garvey, 2022 (26)	USA, Canada, Italy, Spain, Hungary	41	243	0	8
Mu, 2024 (29)	China, South Korea, Hong Kong, Brazil	23	44	0	3
Kahn, 2024 (30)	41, across the world^a^	804	176	0	<1
McGowan, 2024 (31)	Canada, UK, Spain, Denmark, Finland	30	80	28	8
Liraglutide-based RCTs					
Pi-Sunyer, 2015 (21)	27, across the world^a^	191	68	0	2
le Roux, 2017 (23)	27, across the world^a^	191	172	0	2
Svensson, 2019 (24)	Denmark	2	68	58	16
Fogshsgaard, 2023 (27)	Denmark	1	53	1	1
Exenatide-based RCT					
Rosenstock, 2010 (20)	USA	1	28	0	33
SGLT2 inhibitor RCT					
James, 2023 (28)	UK, Sweden	103	12	0	<1
GLP-1 RAs plus SGLT2 inhibitor RCT					
Lundkvist, 2017 (22)	Sweden	1	24	0	14

RCTs, randomized controlled trials; No., number; wks, weeks; T2DM, type 2 diabetes mellitus; USA, United States of America; UK, United Kingdom; mo, months.

a Including all continents.

The incidence of T2DM during the post-intervention follow-up period was assessed in three RCTs (27, 28, 34). The post-intervention duration lasted from 28 to 58 weeks (24, 25, 31) (**Table 1**).

#### 3.1.2 SGLT2 inhibitor RCT

The RCT, which evaluated dapagliflozin, was a multicenter study, including 103 centers in the UK and Sweden (28). It lasted 12 months, and the dropout rate was <1% (28). The design was parallel, and it was published in 2023 (28) (**Table 1**).

#### 3.1.3 GLP-1-RA and SGLT2 inhibitor RCT

The trial that included both exenatide and dapagliflozin was conducted in Sweden in one center (22). The study’s duration was 24 weeks, and the dropout rate was 14% (22). The design was parallel, and it was published in 2017 (22) (**Table 1**).

### 3.2 Characteristics of participants

#### 3.2.1 GLP-1 RA RCTs

The sample size of GLP-1 RA RCTs varied between 103 and 17,604 participants (20, 21, 23–27, 29–31); 35.2% of the participants were men and 64.8% were women (20, 21, 23, 25–27, 29–31). One study did not give information concerning gender (24). Participants’ mean age was 47.5 years (20, 21, 23, 25–27, 29–31). One trial reported the ages of participants ranging between 18 and 65 years (24). Among the participants, 76.7% were Caucasians, 16.9% Asians, 4.4% Black or African-American, 1.6% Hispanic or Latino, and <1% American Indian or Native Hawaiian or Pacific Islander or other (21, 23, 25–27, 29–31). Two RCTs did not report the participants’ race or ethnicity (20, 24) (**Table 2**).

**Table 2:** Characteristics of the participants.

First author’s name, publication year	Sample size (I/C)	Gender %, male/female	Mean age in years (SD)	Race, ethnicity (%)	Risk factors for T2DM		Baseline HbA1c % (SD) (I/C)
					Overall	Probable coexistence	
GLP-1 RA RCTs							
Semaglutide-based RCTs							
Wilding, 2022 (25)	333 (232/101)	32.8/67.2	49 (11.5)	White (75.5), Asian (21), Black or African-American (2.4), Other (0.9)	Overweight/obesity (BMI ≥ 27 kg/m²)	Prediabetes, HY, dyslipidemia, CVD, NAFLD, PCOS	5.7 (0.3)/5.7 (0.3)
Garvey, 2022 (26)	304 (152/152)	22.4/77.6	47.3 (11)	White (93.1), Hispanic or Latino (12.8), Black or African American (3.9), American Indian or Alaska Native (1), Asian (<1), Other (1.3)	Overweight/obesity (BMI ≥ 27 kg/m²)	Prediabetes, HY, dyslipidemia, CAD, NAFLD, PCOS	5.7 (0.3)/5.7 (0.4)
Mu, 2024 (29)	375 (249/126)	55/45	40.5 (11)	Asian (91), White (8), Black or African American (1)	Overweight/obesity (BMI ≥ 27 kg/m²)	Prediabetes, HY, dyslipidemia, CVD, NAFLD, PCOS	6.2 (1.1)/6.3 (1.2)
Kahn, 2024 (30)	17,604 (8,803/8,801)	72.3/27.7	61.6 (8.9)	White (84), Asian (8.2), Black (3.8), Other (3.0), NR (1)	Overweight/obesity (BMI ≥ 27 kg/m²) and CVD	Prediabetes	5.78 (0.34) (overall)
McGowan, 2024 (31)	207 (138/69)	71/29	53 (11)	White (88), Black or African-American (4.0), Asian (4.0), American Indian or Alaska Native (<1), Other (2)	Obesity (BMI ≥ 30 kg/m²) and Prediabetes	HY, dyslipidemia, CVD	5.9 (0.3)/5.9 (0.3)
Liraglutide-based RCTs							
Pi-Sunyer, 2015 (21)	3,731 (2,487/1,244)	21.4/78.4	45.1 (12)	White (85), Black (9.4), Asian (3.6), American Indian or Alaska Native (0.2), Native Hawaiian or other Pacific Islander (0.1), Other (1.5)	Overweight/obesity (BMI ≥ 27 kg/m²)	Prediabetes, HY, dyslipidemia	5.6 (0.4)/5.6 (0.4)
le Roux, 2017 (23)	2,254 (1,505/749)	24/76	47.4 (11.7)	White (84), Black or African-American (10.0), Asian (5), American Indian or Alaska Native (0.3), Native Hawaiian or other Pacific Islander (<0.1), Other (1.3)	Overweight/obesity (BMI ≥ 27 kg/m²) and prediabetes	HY, dyslipidemia	5.8 (0.3)/5.7 (0.3)
Svensson, 2019 (24)	103 (52/51)	NR	Range: 18–65	NR	Overweight/obesity (BMI ≥ 27 kg/m²)^a^ and prediabetes	NR	5.6 (2.6)/5.5 (2.5)
Fogshsgaard, 2023 (27)	105 (50/55)	0/100	37.9 (4.9)	Caucasians 96, non-Caucasians 4; Asian (2.8), Black (1.0)	Overweight/obesity (BMI ≥ 25 kg/m²) and history of GDM	Prediabetes, family history of T2DM	5.1 (2.2) (overall)
Exenatide-based RCT							
Rosenstock, 2010 (20)	152 (73/79)	18/82	46 (12)	NR	Obesity (BMI ≥ 30 kg/m²)	Prediabetes	NR
SGLT2 inhibitor RCT							
James, 2023 (28)	4,017 (2,019/1,998)	79.9/20.1	62.9 (10.8)	White (94.5), Asian (2.9), Black (<1), Other (1.9), NR 3 (<1)	CAD^b^	HY, CVD	5.7 (0.58)/5.7 (0.51)
GLP-1 RAs plus SGLT2 inhibitor RCT							
Lundkvist, 2017 (22)	50 (25/25)	38.7/61.3	51.7 (12.6)	NR	Obesity (BMI ≥ 30 kg/m²)	Prediabetes, HY, dyslipidemia	5.6 (0.35)/5.6 (0.30)

GLP-1 RAs, glutagone-like-peptide-1 receptor agonists; RCTs, randomized controlled trials; I, intervention; C, comparator; SD, standard deviation; T2DM, type 2 diabetes mellitus; HbA1c, hemoglobin A1c; BMI, body mass index; kg, kilogram; m, meter; HY, hypertension; CVD, cardiovascular disease; NAFLD, non-alcoholic fatty liver disease; PCOS, polycystic ovary syndrome; NR, non-reported; GDM, gestational diabetes mellitus; CAD, cardiovascular disease.

a Schizophrenia spectrum disorder treated with clozapine or olanzapine.

b Clinically stable patients hospitalized for myocardial infarction and with impaired left-ventricle function without known diabetes or established heart failure.

Overweight or obesity was the common diabetes risk factor that affected all participants (20, 21, 23–27, 29–31). Five RCTs reported additional diabetes risk factors concerning all patients (23, 24, 27, 30, 31): three of them reported prediabetes (23, 24, 31), one history of GDM (27), and another CVD (30). Probable co-existence of diabetes risk factors affecting some of the individuals was reported in nine RCTs (20, 21, 23, 25–27, 29–31). They included prediabetes (20, 21, 25–27, 29, 30), hypertension (21, 23, 25, 26, 29, 31), dyslipidemia (21, 23, 25, 26, 29, 31), CVD (31), NAFLD (25, 26, 29), PCOS (25, 26, 29), and family history of T2DM (27). The baseline HbA1c in the intervention arms varied from 5.6% (21, 24) to 6.2% (29) and between 5.5% (24) and 6.3% (29) in the control groups. Two studies reported the overall baseline HbA1c of the participants, independent of the assigned group (27, 30). One study did not provide data concerning HbA1c (20) (**Table 2**).

#### 3.2.2 SGLT2 inhibitor RCT

The RCT of dapagliflozin included 4,017 participants, mostly men (79.9%) with a mean age of 62.9 years (28). Of the participants, 94.5% were Caucasians, 2.9% Asians, 1.9 other, and <1% Black (28). The main diabetes risk factor was coronary artery disease (CAD) (28). Particularly, participants were non-diabetic patients with myocardial infarction (MI), clinically stable, and without heart failure, and some of them had already established CAD or HY (28). The baseline HbA1c was 5.7% in both intervention and control groups (**Table 2**).

#### 3.2.3 GLP-1 RA and SGLT2 inhibitor RCT

The RCT of exenatide and dapagliflozin included 50 participants (22). Most of them were women (61.3%) with a mean age of 51.7 years (22). Obesity was the most common diabetes risk factor, while prediabetes, HY, and dyslipidemia affected a number of individuals (22). The baseline HbA1c was 5.6% in patients assigned either to the experimental or placebo group (**Table 2**).

### 3.3 Characteristics of interventions

#### 3.3.1 GLP-1 RA RCTs: semaglutide-based RCTs

##### 3.3.1.1 Semaglutide

All studies evaluated a dosage of semaglutide at 2.4 mg once weekly (25, 26, 29–31). A titration strategy every 4 weeks was followed in all trials, reaching the final dosage of 2.4 mg (25, 26, 29–31). However, the initial dose was not the same, with four RCTs reporting 0.25 mg (25, 26, 29, 31) and one RCT reporting 0.6 mg (30). The period of administration varied between 44 and 152 weeks (25, 26, 29–31). However, adherence to pharmaceutical interventions was not reported in any RCT (25, 26, 29–31) (**Supplementary Table S2**).

##### 3.3.1.2 Lifestyle, placebo

In four trials, all participants received counseling on lifestyle modification, including increased PA and reduced calorie diet (25, 26, 29, 31). One study adopted local guidelines for standard care after the end of pharmaceutical and lifestyle interventions (31). In-person or clinical visits, telephone calls, diaries, and mobile applications were applied in two studies to assess adherence to lifestyle interventions (26, 29), while assessment of adherence was not reported in three studies (25, 30, 31). One study did not report any lifestyle intervention, and semaglutide was compared to placebo (30). Comparators in all studies received placebo (25, 26, 29–31) (**Supplementary Table S2**).

#### 3.3.2 GLP-1 RA RCTs: liraglutide-based RCTs

##### 3.3.2.1 Liraglutide

Daily doses of 1.8 mg in two RCTs (24, 27) and 3.0 mg in two other studies (21, 23) were evaluated for liraglutide. A weekly titration followed an initial dosage of 0.6 mg in all studies (21, 23, 24, 27). The duration varied between 16 and 160 weeks (21, 23, 24, 27). The studies did not report assessment of adherence to pharmaceutical interventions (21, 23, 24, 27) (**Supplementary Table S2**).

##### 3.3.2.2 Lifestyle, placebo

Two RCTs reported the adoption of lifestyle modification for all patients (21, 23). They consisted of increased PA and reduced nutrition consumption, including macronutrient distribution: 30% of energy from fat, 20% from protein, and 50% from carbohydrate (21, 23). They both lasted 12 weeks more than the medicine intervention, totaling 68 and 172 weeks, respectively (21, 23). Food diaries and pedometers were used to assess adherence (21, 23). Two studies evaluated liraglutide versus placebo (24, 27). The participants in the control groups of all studies received placebo (21, 23, 24, 27) (**Supplementary Table S2**).

#### 3.3.3 GLP-1 RA RCTs: exenatide-based RCT

The intervention of exenatide lasted 24 weeks (20). The dosage was 10 mg equally divided twice daily, after an initial dose of 5 mg, which lasted 4 weeks (20). All participants also received lifestyle counseling, and the control group received placebo (20) (**Supplementary Table S2**).

#### 3.3.4 SGLT2 inhibitor intervention

The intervention included 10 mg of dapagliflozin daily for 12 months (28). All participants received standard therapy for CAD, and comparators also received placebo (28). (**Supplementary Table S2**).

#### 3.3.5 Combined GLP-1 RA and SGLT2 inhibitor intervention

The participants in the intervention arm received 10 mg of dapagliflozin daily and 2 mg of exenatide once weekly for 24 weeks (22). The number of returned products and self-reports was used to assess the intervention’s adherence (22) (**Supplementary Table S2**).

### 3.4 Primary outcomes

#### 3.4.1 T2DM outcome assessment and diagnosis

##### 3.4.1.1 GLP-1 RA RCTs

The outcome of T2DM was reported as primary in three GLP-1 RA studies (23, 24, 30). Additional primary outcomes were changes in body weight (24) and glycemic control (23, 30). T2DM was a secondary outcome in the other seven trials (20, 21, 25–27, 29, 31). Their primary outcomes were changes in body weight (20, 21, 25, 26, 29, 31), glycemic control (20, 27, 31), and changes in cardiometabolic risk factors (25). The laboratory tests of diabetes diagnosis included HbA1c, FPG, 2- or 4-h 75 g OGTT, and random glucose measurement in patients with classic symptoms of hyperglycemia (20, 21, 23–27, 29–31) (**Supplementary Table S3**).

##### 3.4.1.2 SGLT2 inhibitor RCT

The incidence of T2DM was a primary outcome in addition to mortality, cardiovascular disease, hospitalization, and changes in body weight in the study of dapagliflozin (28). The diagnosis of T2DM was set by HbA1c (28) (**Supplementary Table S3**).

##### 3.4.1.3 Combined GLP-1 RA and SGLT2 inhibitor RCT

In the study of exenatide and dapagliflozin, T2DM was a secondary outcome with changes in body weight, glycemic control, and systolic blood pressure (SBP) comprising the primary outcomes (22). The diagnostic modalities included FPG, HbA1c, and 2-h 75 gram (g) OGTT (22) (**Supplementary Table S3**).

#### 3.4.2 Effectiveness of interventions in preventing T2DM

##### 3.4.2.1 Overall effectiveness of GLP-1 RAs

**Figure 2:**
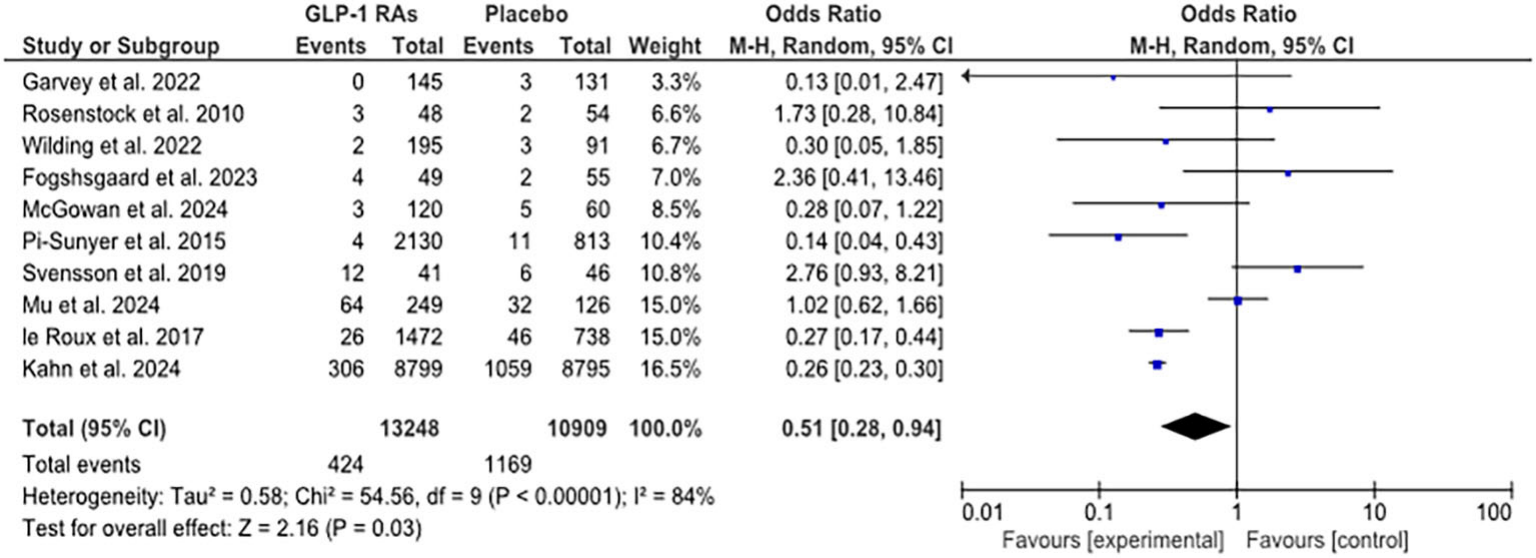
Forest plot of the meta-analysis of GLP-1-RA-based RCTs.

A total of 24,157 (13,248 in the intervention and 10,909 in the control groups) high-risk participants were analyzed for T2DM in GLP-1 RA RCTs (**Supplementary Table S3**; **Figure 2**). Combining studies in MA, there was a significant heterogeneity (*Q* 54.56; *P*-value < 0.0001; *I*² 84%; 95% CI 74%, 89%) (**Figure 2**). A protective antidiabetic effect of GLP-1 RAs was found in high-risk patients (OR 0.51; 95% CI 0.28, 0.94; *P*-value 0.03) (**Figure 2**).

##### 3.4.2.2 Type of GLP-1 RA-based subgroup analysis

Exploring the effectiveness of GLP-1 RAs according to the type of drugs, there was a statistically significant effect for semaglutide (OR 0.38; 95% CI 0.16, 0.94; *P*-value < 0.0001), but not for liraglutide (OR 0.64; 95% CI 0.16, 2.53; *P*-value 0.53) or exenatide (OR 1.73; 95% CI 0.28, 10.84; *P*-value 0.56). However, the test of difference was non-significant (*P*-value 0.34) (**Supplementary Table S4**, **Supplementary Figure S1**).

##### 3.4.2.3 Semaglutide-based subgroup and sensitivity analyses

Semaglutide demonstrated an effect in multicenter studies that were performed either across the world (OR 0.26; 95% CI 0.23, 0.30; *P*-value < 0.00001) or in North America and Europe (OR 0.24; 95% CI 0.06, 0.90; *P*-value 0.03) (**Supplementary Figure S2**). Additionally, for subgroup analysis based on studies assessing the incidence of diabetes during the intervention and post-intervention period, the result was significant for post-intervention duration (OR 0.29; 95% CI 0.09, 0.91; *P*-value 0.03) (**Supplementary Figure S3**). No effect was found in gender-based subgroup analysis (**Supplementary Figure S4**). Performing analyses based on the participants’ mean age, a significant result was found for patients’ mean age more than 50 years (OR 0.26; 95% CI 0.23, 0.30; *P*-value < 0.00001) (**Supplementary Figure S5**). A significant effect was also found in the analysis considering CVD as the main factor, in addition to obesity and diabetes risk factor (OR 0.26; 95% CI 0.23, 0.30; *P*-value < 0.00001) (**Supplementary Figure S6**). Subgroup analysis concerning the intervention’s duration reported effectiveness for interventions lasting more than 100 weeks (OR 0.26; 95% CI 0.23, 0.30; *P*-value < 0.00001) (**Supplementary Figure S7**). Finally, significance was found for studies assessing T2DM as a primary outcome (OR 0.26; 95% CI 0.23, 0.30; *P*-value < 0.00001) (**Supplementary Figure S8**). The semaglutide-based subgroup test of differences was statistically significant in the analyses concerning similar countries and regions (**Supplementary Figure S2**) and non-significant in the rest of the analyses (**Supplementary Figures S3–S8**, **Supplementary Table S4**).

Sensitivity semaglutide-based analyses affected the results for a study with the largest sample size (OR 0.50; 95% CI 0.19, 1.29; *P*-value 0.15) (**Supplementary Table S4**, **Supplementary Figure S9**), for studies with post-intervention follow-up duration (OR 0.43; 95% CI 0.13, 1.43; *P*-value 0.17) (**Supplementary Table S4**, **Supplementary Figure S10**), and for studies with a dropout rate more than 5% (OR 0.51; 95% CI 0.13, 1.90; *P*-value 0.31) (**Supplementary Table S4**, **Supplementary Figure S11**).

##### 3.4.2.4 Liraglutide-based subgroup and sensitivity analyses

A protective effect was revealed in liraglutide-based studies that were performed worldwide (OR 0.24; 95% CI 0.14, 0.40; *P*-value < 0.00001), but not in Europe (Denmark) (**Supplementary Figure S12**). Effectiveness was also found in studies without follow-up duration (OR 0.24; 95% CI 0.14, 0.40; *P*-value < 0.00001) (**Supplementary Figure S13**). Liraglutide was also effective in studies with participants’ mean age more than 40 years (OR 0.24; 95% CI 0.14, 0.40; *P*-value < 0.00001) (**Supplementary Figure S14**). Daily dosage was effective at 3.0 mg (OR 0.24; 95% CI 0.14, 0.40; *P*-value < 0.00001) (**Supplementary Figure S15**). Interventions’ duration was significant when it lasted more than 55 weeks (OR 0.24; 95% CI 0.14, 0.40; *P*-value < 0.00001) (**Supplementary Figure S16**). The test of difference was significant in those subgroup analyses (**Supplementary Figures S12–S16**). Finally, there were neither statistically significant results nor significant test of difference for studies assessing T2DM either as a primary or a secondary outcome (**Supplementary Figure S17**, **Supplementary Table S4**).

Liraglutide-based sensitivity analyses were performed to assess the effect of the study with the largest sample size (**Supplementary Table S4**, **Supplementary Figure S18**), post-intervention follow-up duration (**Supplementary Table S4**, **Supplementary Figure S19**), and dropout rate more than 10% (**Supplementary Table S4**, **Supplementary Figure S20**); however, only studies with post-intervention follow-up significantly affected the result (OR 0.24; 95% CI 0.14, 0.40; *P* < 0.00001) (**Supplementary Table S4**, **Supplementary Figure S19**).

##### 3.4.2.5 Effectiveness of SGLT2 inhibitor

One study assessing the SGLT2 inhibitor dapagliflozin reported a lower incidence of T2DM (hazard ratio 0.53; 95% CI 0.36, 0.77) (**Supplementary Table S3**).

##### 3.4.2.6 Effectiveness of combined GLP-1 RA and SGLT2 inhibitor

The study evaluating both exenatide and dapagliflozin reported one case of diabetes in the placebo group and none in the intervention group (**Supplementary Table S3**).

### 3.5 Secondary outcomes

#### 3.5.1 Changes in body weight

##### 3.5.1.1 Semaglutide RCTs

All semaglutide-based RCTs assessed weight reduction (25, 26, 30, 31, 39). The sample size varied between 180 (31) and 17,594 patients (30). The mean difference in weight loss in the intervention arm varied between 6.6 (25) and 16.1 kilograms (kg) (26) (**Supplementary Table S5**).

##### 3.5.1.2 Liraglutide RCTs

Similarly, all studies of liraglutide considered weight loss (21, 23, 24, 27). The lower sample size included 73 participants (24) and the largest 2,943 (21). Mean weight loss ranged between 4.9 (27) and 8.4 kg (21) in the liraglutide group (**Supplementary Table S5**).

##### 3.5.1.3 Exenatide RCT

The trial reported weight loss of 5.1 and 1.6 kg in the intervention and control arms, respectively (20) (**Supplementary Table S5**).

##### 3.5.1.4 SGLT2 inhibitor RCT

Patients who received dapagliflozin had a mean weight loss of 1.41 kg compared to 0.24 in the placebo group (28) (**Supplementary Table S5**).

##### 3.5.1.5 Combined GLP-1 RA and SGTL2 inhibitor RCT

The combination of exenatide and dapagliflozin led to a mean 4.48 kg weight loss compared to 0.35 kg in the placebo group (22) (**Supplementary Table S5**).

##### 3.5.1.6 Comparison of body weight changes in GLP-1 RAs

All eligible trials were included in the MA (20, 21, 23–27, 29–31). There were 13,240 patients in the intervention arm who had a significant weight loss compared to 10,903 patients in the placebo arm (mean difference −6.35; 95% CI −8.63, −4.07; *P*-value < 0.00001). Subgroup analyses based on the type of GLP-1 RAs revealed a greater weight loss by semaglutide (mean difference −8.82; 95% CI −11.47, −6.16; *P*-value < 0.00001). Liraglutide was the next drug in weight loss (mean difference −4.06; 95% CI −5.47, −2.64; *P*-value < 0.00001) and exenatide the last (mean difference −3.50; 95% CI −3.69, −3.31; *P*-value < 0.00001). The administration of any drug led to a significant weight loss, and differences in the analysis were significant (*P*-value 0.0004) (**Supplementary Table S6**).

#### 3.5.2 Changes in BMI

##### 3.5.2.1 Semaglutide RCTs

Three studies provided data for BMI (25, 26, 29). Two trials did not report information (30, 31). Their sample size included 276 (26), 286 (25), and 375 patients (29). The intervention arms had a mean reduction of BMI between 2.6 and 4.1 kg/meter (m)² (**Supplementary Table S5**).

##### 3.5.2.2 Liraglutide RCTs

Two RCTs reported changes in BMI (23, 24). The RCT with the lower sample size of 73 patients reported a lower mean decrease in BMI of 0.7 kg/m² (24) compared to the RCT with the largest sample size of 2,210 participants and mean reduction of 2.4 kg/m² for patients in the intervention groups (23). Two studies did not give information (21, 27) (**Supplementary Table S5**).

##### 3.5.2.3 Exenatide RCT

There were no available data (20) (**Supplementary Table S5**).

##### 3.5.2.4 SGLT2 inhibitor RCT

Similarly, no reports were available (28) (**Supplementary Table S5**).

##### 3.5.2.5 Combined GLP-1 RA and SGLT2 inhibitor RCT

This study did not include the outcome of BMI (22) (**Supplementary Table S5**).

##### 3.5.2.6 Comparison of changes in BMI in GLP-1 RA RCTs

Five eligible RCTs were included in the analysis (23–26, 29). Three of them assessed semaglutide (25, 26, 29) and two evaluated liraglutide (23, 24). The pooled analysis included 2,094 participants in the intervention group and 1,126 in the control group and demonstrated a significant reduction of BMI with GLP-1 RAs (mean difference −2.46; 95% CI −3.17, −1.76; *P*-value < 0.00001). Semaglutide and liraglutide were both significant, but semaglutide was more effective than liraglutide (mean difference −3.23; 95% CI −3.94, −2.52; *P*-value < 0.00001 and mean difference −1.29− 95% CI –2.08, –0.51; *P*-value 0.001, respectively). The test of difference was significant (*P*-value 0.0003) (**Supplementary Table S6**).

#### 3.5.3 Changes in HbA1c

##### 3.5.3.1 Semaglutide RCTs

Five studies assessed changes in HbA1c (25, 26, 30, 31, 39). Patients who received semaglutide had lower mean HbA1c compared to the placebo from 0.1% (25, 31) to 0.80% (29) (**Supplementary Table S5**).

##### 3.5.3.2 Liraglutide RCTs

All liraglutide-based studies assessed also HbA1c (21, 23, 24, 27). Three trials demonstrated a mean reduction of 0.30% (21), 0.35% (23), and 2.6% (27). Notably, one study reported an increase of HbA1c: 2.3% in the intervention group and 2.2% in the control group (24) (**Supplementary Table S5**).

##### 3.5.3.3 Exenatide RCTs

There was no information provided (20) (**Supplementary Table S5**).

##### 3.5.3.4 SGLT2 inhibitor RCT

Also, HbA1c was not reported (28) (**Supplementary Table S5**).

##### 3.5.3.5 GLP-1 RA and SGLT2 inhibitor RCTs

Patients under exenatide and dapagliflozin had a reduced mean HbA1c of 0.36% and patients in the placebo had 0.15% (22) (**Supplementary Table S5**).

##### 3.5.3.6 Comparison of changes in HbA1c in GLP-1 RA RCTs

All semaglutide-based (25, 26, 30, 31, 39) and all liraglutide-based RCTs (21, 23, 24, 27) were combined in the analysis. There were 13,192 and 10,849 participants in the intervention and control groups. The decrease of HbA1c was significant (mean difference −0.37; 95% CI −0.48, −0.27; *P*-value < 0.00001). Semaglutide and liraglutide were effective, and the reduction was more pronounced in liraglutide compared to semaglutide (mean difference −0.41; 95% CI −0.60, −0.22; *P*-value < 0.0001 and mean difference −0.36; 95% CI −0.50, −0.22; *P*-value < 0.00001, respectively). However, the test of difference was non-significant (*P*-value 0.66) (**Supplementary Table S6**).

#### 3.5.4 Changes in FPG

##### 3.5.4.1 Semaglutide RCTs

Three studies reported assessment of FPG values (26, 29, 31). A greater mean reduction was found for studies with the largest sample size: 1 millimole (mmol)/liter (L) for the study with 375 participants (29), 0.4 mmol/L for the study with 276 participants (26), and 0.3 mmol/L for the study with 180 participants (31). FPG was not reported in two RCTs (25, 30) (**Supplementary Table S5**).

##### 3.5.4.2 Liraglutide RCTs

All trials assessed FPG (21, 23, 24, 27). For semaglutide, a greater mean reduction was observed for the studies with the largest sample size: 0.40 mmol/L in the study with 2,943 participants (21), 0.37 mmol/L in the study with 2,210 participants (23), and 0.27 mmol/L in the study with 104 participants (27). Notably, the study with the lower sample size of 73 participants reported an increase of mean FPG of 1.02 mmol/L for patients under liraglutide (24) (**Supplementary Table S5**).

##### 3.5.4.3 Exenatide RCT

The exenatide-based RCT did not provide data (20) (**Supplementary Table S5**).

##### 3.5.4.4 SGLT2 inhibitor RCT

Also, the dapagliflozin-based study did not give information (28) (**Supplementary Table S5**).

##### 3.5.4.5 Combined GLP-1 RA and SGLT2 inhibitor RCT

Patients who received exenatide and dapagliflozin had a reduction of mean FPG of 0.41 mmol/L and patients in the placebo arm had a mean increase of 0.21 mmol/L (22) (**Supplementary Table S5**).

##### 3.5.4.6 Comparison of changes in FPG in GLP-1 RA RCTs

For FPG, 4,198 patients in the GLP-1 RA groups and 1,963 in the control groups were analyzed. The analysis included three semaglutide-based studies (26, 29, 31) and all four liraglutide-based studies (21, 23, 24, 27). GLP-1 RAs significantly decreased FPG compared to placebo (−0.42; 95% CI −0.62, −0.21; *P*-value < 0.0001). Semaglutide and liraglutide were significant and semaglutide achieved a greater reduction against liraglutide (mean difference −0.55; 95% CI −0.88, −0.22; *P*-value 0.001 and −0.39; 95% CI −0.46, −0.33; *P*-value < 0.00001, respectively). However, no significant test of difference was found (*P*-value 0.35) (**Supplementary Table S6**).

#### 3.5.5 Changes in 2-h OGTT

##### 3.5.5.1 Semaglutide RCTs

There were no available data in those RCTs (25, 26, 30, 31, 39) (**Supplementary Table S5**).

##### 3.5.5.2 Liraglutide RCTs

One study reported a reduction of 2-h mean plasma glucose during the 2-h OCTT by 1.6 mmol/L with liraglutide versus 0.2 mmol/L with placebo (23) (**Supplementary Table S5**).

##### 3.5.5.3 Exenatide RCT

Glucose changes during OGTT were not reported (20) (**Supplementary Table S5**).

##### 3.5.5.4 SGLT2 inhibitor RCT

Also, no information was available for this outcome (28) (**Supplementary Table S5**).

##### 3.5.5.5 Combined GLP-1 RA and SGLT2 inhibitor RCT

The 2-h mean plasma glucose during the 2-h OGTT was decreased by 1.57 mmol/L in the intervention arm and 0.88 mmol/L in the placebo arm (22) (**Supplementary Table S5**).

#### 3.5.6 Adverse events

##### 3.5.6.1 Semaglutide RCTs

The majority of gastrointestinal disorders or symptoms related to semaglutide’s intervention were mild (26, 29, 31). One study reported that they affected more than 10% of participants (26) and another study reported more than 5% (29). Gallbladder and hepatobiliary disorders were rare (26, 29). Acute pancreatitis was reported in another study including two cases in the intervention group compared to the placebo group (31). Patients receiving semaglutide or not were similarly affected with infections; musculoskeletal, psychiatric, and nervous system disorders; CVD events; and neoplasms, indicating no association with intervention (26, 29, 31). Two RCTs did not give information for adverse events (25, 30) (**Supplementary Table S7**).

##### 3.5.6.2 Liraglutide RCTs

Two liraglutide-based RCTs reported more adverse events concerning mild gastrointestinal symptoms and decreased appetite affecting more than 5% of participants (21, 23). Serious gastrointestinal disorders including choledocholithiasis and cholecystitis affected more than 0.2% and 0.4%, respectively (21, 23). One study reported mild acute pancreatitis in four patients receiving liraglutide versus none in placebo (21), and another study reported elevated lipase levels in 10 patients in the intervention group compared to four in the placebo group (23); however, there was no report of pancreatitis (23). Injection-site symptoms were reported for some patients in both groups (21, 23). Adverse events including musculoskeletal disorders, infections, headache, CVD events, and neoplasms were not associated with liraglutide (21, 23). Two RCTs did not report adverse events (24, 27) (**Supplementary Table S7**).

##### 3.5.6.3 Exenatide RCT

Mild to moderate gastrointestinal symptoms were reported in the trial involving exenatide (20) (**Supplementary Table S7**).

##### 3.5.6.4 SGLT2 inhibitor RCT

There was no increase of side effects related to the administration of dapagliflozin (28) (**Supplementary Table S6**).

##### 3.5.6.5 GLP-1 RA and SGLT2 inhibitor RCT

Decreased appetite and mild gastrointestinal symptoms were possibly associated with the complex intervention of exenatide and dapagliflozin (22). Additionally, injection-site disorders affected participants in both experimental and control groups (22). Adverse events such as hypotension and infections were not related to the intervention (22). Both the intervention and control groups had equal adverse events (22) (**Supplementary Table S7**).

##### 3.5.6.6 Comparison of major adverse events in GLP-1 RA RCTs

The safety analysis included the major adverse events in GLP-1 RAs and placebo arms. Five trials reported major adverse events (21, 23, 26, 29, 31) (**Supplementary Table S7**). Three of them assessed semaglutide (26, 29, 31) and two liraglutide (21, 23) (**Supplementary Table S7**). The semaglutide-based studies reported a total of 37 patients who had major adverse events in the intervention group versus 32 in the placebo group (26, 29, 31); the liraglutide-based studies reported 120 versus 45, respectively (21, 23) (**Supplementary Table S7**). The pooled analysis did not find significance between the compared arms (OR 1.01; 95% CI 0.76, 1.35; *P*-value 0.95) (**Supplementary Table S6**). Finally, the subgroup analyses based on the type of GLP-1 RA remained non-significant for semaglutide (OR 0.73; 95% CI 0.44, 1.22; *P*-value 0.23) and liraglutide (OR 1.17; 95% CI 0.83, 1.66; *P*-value 0.37) (**Supplementary Table S6**).

## 4 Risk of bias and quality of evidence

### 4.1 Risk of bias

#### 4.1.1 GLP-1 RA RCTs

Overall risk of bias was low for two studies (27, 29). Some concern was raised from five RCTs (24–26, 30, 31). Bias in the randomization process could not be excluded in all studies (24–26, 30, 31), and some issues were found in the measurement of the outcome in four of them (24–26, 30). Finally, three trials were judged as having an overall high risk of bias (20, 21, 23). They all had high risk due to missing outcome data (20, 21, 23). Moreover, bias arose from deviations from intended interventions in one study (20) (**Supplementary Table S8**).

#### 4.1.2 SGLT2 inhibitor RCT

The RCT involving dapagliflozin was considered low risk for any potential bias (28) (**Supplementary Table S8**).

#### 4.1.3 GLP-1 RA and SGLT2 inhibitor RCT

The study evaluating exenatide and dapagliflozin was assessed as having an overall high risk of bias (22). Biases arising from the randomization process and missing outcome data were considered high (22) (**Supplementary Table S8**).

### 4.2 Publication bias

**Figure 3:**
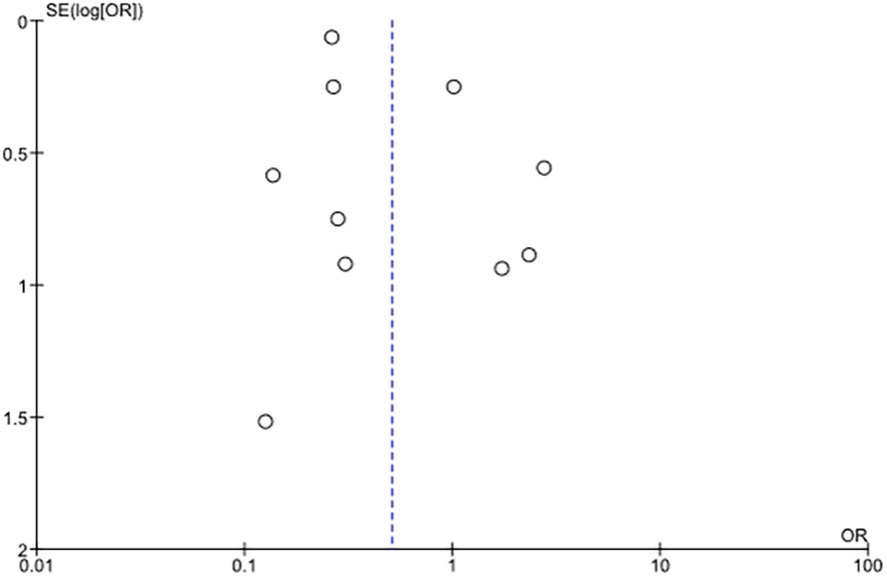
Forest plot of the meta-analysis of GLP-1 RA RCTs.

Considering the MA including GLP-1 RA trials (21, 23, 25–27, 29–31), the Egger’s test statistic *P*-value was 0.153, indicating the absence of publication bias. However, the funnel plot of MA does not show a symmetric distribution of studies (**Figure 3**).

### 4.3 Quality of evidence

The overall quality of evidence was low for studies evaluating the effectiveness of GLP-1 RAs (21, 23, 25–27, 29–31). The factors associated with low quality of evidence are inconsistency due to heterogeneity and publication bias (**Table 3**).

**Table 3:** GRADE evaluation of overall evidence of studies according to analyses for GLP-1 RAs.

GLP-1 RAs compared to placebo for T2DM prevention					
Outcome	No. of participants (studies)	Quality of tde evidence (GRADE)	Relative effect (95% CI)	Anticipated absolute effects	
				Risk witd placebo	Risk difference witd GLP-1 RAs (95% CI)
T2DM	24,157 (10 studies)	⊕⊕⊝⊝ LOW due to inconsistency, publication bias	OR 0 (0.28 to 0.94)	Study population	
				107 per 1,000	107 fewer per 1,000 (from 6 fewer to 75 fewer)
				Moderate	
*Tde basis for tde assumed risk (e.g., tde median control group risk across studies) is provided in tde footnotes. Tde corresponding risk (and its 95% confidence interval) is based on tde assumed risk in tde comparison group and tde relative effect of tde intervention (and its 95% CI). CI, confidence interval; OR, odds ratio.					
GRADE Working Group grades of evidence High quality: Furtder research is very unlikely to change our confidence in tde estimate of effect. Moderate quality: Furtder research is likely to have an important impact on our confidence in tde estimate of effect and may change tde estimate. Low quality: Furtder research is very likely to have an important impact on our confidence in tde estimate of effect and is likely to change tde estimate. Very low quality: We are very uncertain about tde estimate.					

GLP-1 RAs, glucagon-like peptide-1 receptor agonists; T2DM, type 2 diabetes mellitus.

## 5 Discussion

In the present study, we found that GLP-1 RAs were associated with a reduced risk of T2DM in high-risk patients. The main common diabetes risk factor was overweight or obesity. A 62% risk reduction for T2DM was found when semaglutide was used, while this was not identified with liraglutide and exenatide. Semaglutide was found effective in patients from all continents and from North America and Europe, aged more than 50 years and had CVD as the main risk factor in addition to overweight/obesity, receiving the drug for more than 100 weeks. However, the liraglutide-based subgroup analyses support the effectiveness in high-risk adults, with a mean age over 40 years, at daily dosage of 3.0 mg, after 55 weeks of administration, and without post-intervention duration. Liraglutide was mainly assessed in women. Heterogeneity was significant, and the associated factors include the following: 1) different countries where studies involving semaglutide were performed; 2) the study with the largest sample size, the studies with post-intervention duration, and the studies with a dropout rate more than 5% in semaglutide-based sensitivity analyses; 3) subgroup liraglutide-based analyses comparing countries of studies’ performance, participant’s mean age, daily dosage, intervention’s duration, and post-intervention duration; and 4) the studies with post-intervention duration in liraglutide-based sensitivity analyses. Large heterogeneity contributes to inconsistency of the results. Publication bias is also possible. Those factors downgrade the overall quality of evidence to low, which means that further investigation is needed to support our estimations. Finally, the RCT that assessed dapagliflozin in patients with CAD reported a protective effect for T2DM, while fewer diabetes cases were reported for both dapagliflozin and exenatide in patients with obesity versus placebo. Our study demonstrated the effectiveness of GLP-1 RAs in reducing T2DM in high-risk patients, contrariwise to the previous MA that reported no effect for the same patients (11). Two other MAs, including only three (12) and six RCTs (13), reported a preventive antidiabetic effect of GLP-1 RAs in patients with obesity and prediabetes (12, 13); however, they both failed to explain heterogeneity (12, 13). Additionally, one of them included *post hoc* analyses and non-eligible RCT, probably affecting the overall effect (14). Thus, our MA may be considered the first that demonstrate the effectiveness of GLP-1 RAs in preventing T2DM in high-risk patients independent of their glycemic status. In our MA, we included all available and most recent RCTs evaluating only GLP-1 RAs’ interventions, reaching the largest sample size that has ever been estimated.

As our study has included RCTs considering overweight or obesity as the main diabetes risk factor, our results may support a significant effect of GLP-1 RAs for preventing T2DM in these patients. This effect is probably associated with the drugs’ mechanisms of action. GLP-1 is naturally produced by the small intestine after meals, increasing insulin’s and decreasing glucagon’s pancreatic secretion, slowing gastric emptying, and reducing appetite (22, 24). GLP-1 RAs enhance the natural insulin stimulation, glucagon’s secretion reduction, and delayed gastric emptying, leading to increased satiety, regulated appetite, and reduced hunger and food consumption, thus contributing to weight loss (2).

The analysis, according to the type of GLP-1 Ras, demonstrated that semaglutide was the most effective drug. Probably, this action is explained by the finding of semaglutide being the most effective anti-obesity GLP-1 RA (32). However, the assessed dosage of semaglutide was 2.4 mg in all trials (25, 26, 30, 31, 39). Investigating the protective antidiabetic action of lower doses might be challenging. Additionally, we found statistically significant results for 3.0 mg of the daily dosage of liraglutide, which is equal to the dosage for obesity management (7). Weight loss may decrease the incidence of diabetes (20, 23). Particularly, a weight reduction of 5%–10% significantly reduces obesity-related compilations (21), including T2DM, hypertension, dyslipidemia, CVD, and increased mortality and cost (25, 29), and improves quality of life (21).

In our study, it was demonstrated that high-risk patients may have a protective effect for diabetes after 100 weeks of semaglutide administration and after 55 weeks of liraglutide administration. Although health behavior interventions are cost-effective for DM prevention (33), the weight reduction in behavioral interventions is often clinically non-significant (26). Additionally, it is difficult to be adopted over time and maintain weight loss (20, 21, 26). Pharmacotherapy is indicated as an adjunct to lifestyle interventions for maintaining long-term weight loss and avoid rebound of weight gain after drug cessation that may occur despite ongoing lifestyle modifications (25). Thus, the long-term need for pharmacotherapy for treating obesity may explain our result for the significant antidiabetic action of semaglutide and liraglutide after 100 and 55 weeks of administration, respectively.

GLP-1 and SGLT2 inhibitors were found to significantly reduce T2DM in patients with CVD. In detail, dapagliflozin reported significant results for diabetes prevention in patients with MI, while semaglutide was effective in preventing T2DM in patients with CVD in addition to overweight/obesity. SGLT2 inhibitors reduce the reabsorption of glucose in the kidneys, excrete calories, and induce mild diuresis, leading to weight loss (22). They improve myocardial structure, function, and sodium–hydrogen exchange (34), offering cardiometabolic benefit in patients with T2DM and/or heart failure (28). However, SGLT2 inhibitors have no reported potential cardiometabolic benefits in non-diabetic overweight or obese adults, despite the significant weight reduction (9, 10). In patients without diabetes, several studies support the cardiometabolic benefits of GLP-1 RAs (35–37). Liraglutide may reduce systolic blood pressure (35), while semaglutide may reduce cardiovascular events and mortality in non-diabetic patients with obesity and CVD (36). Furthermore, GLP-1 RAs may improve insulin resistance and ameliorate the lipidemic profile in adults without T2DM (37).

In our study, we found that GLP-1 RAs decreased weight and BMI in high-risk patients for T2DM. Either semaglutide or liraglutide was significant. However, semaglutide was the most effective drug for weight and BMI reduction. This action may explain the protective action in T2DM development. Additionally, dapagliflozin reported weight loss, but the reduction was smaller than GLP-1 Ras. Moreover, GLP-1 RAs significantly improved HbA1c and FPG. Interestingly, GLP-1 RAs were safe, and this is an important finding for their potential use for the prevention of T2DM. In the same line with our findings, a previous SR of 112 RCTs reported a non-significant long-term increase of major adverse events related to GLP-1 RAs (38); however, a recent MA demonstrated mild gastrointestinal adverse events and hypoglycemia (39).

It should be noted that the RCT of Kahn et al. (30) was the study with the most significant impact on our findings. It provided the largest sample size with 17,594 participants (30), contributing to significant results for semaglutide and GLP-1 RAs overall. Sensitivity analysis found that semaglutide fails to prevent T2DM without this trial, explaining heterogeneity. Biases due to deviations from intended interventions, missing outcome data, and selection of the reported results were judged as low. However, there were some concerns arising from the randomization process and measurement of the outcome due to a lack of information on whether baseline differences between compared arms suggested a problem with randomization and whether outcome assessors were aware of the received interventions by participants, respectively (30). Further large and high-quality RCTs such as the one by Kahn et al. are needed to strengthen the effectiveness of GLP-1 RAs in preventing T2DM.

### 5.1 Strengths and limitations

The present study has several strengths. To our knowledge, it provides the most updated evidence on the protective effects of GLP-1 RAs in preventing T2DM. Analyses based on the type of GLP-1 RAs and the specific characteristics of studies and participants may indicate individual-tailored interventions. The inclusion of trials that have recruited patients from geographically diverse regions enhances the generalizability of the findings. Additionally, the assessment of effectiveness after intervention revealed effectiveness for semaglutide lasting between 28 and 58 weeks. The present study has several limitations as well. The patients’ main diabetes risk factor was obesity. Thus, an overall assessment of GLP-1 RAs in other high-risk groups could not be performed. Moreover, participants may have additional clinical conditions confounding the results. Men are underestimated in the studies involving liraglutide, and there was only one available study assessing exenatide. Heterogeneity was large; however, we identified sources of heterogeneity. Additionally, the overall quality of evidence for the effectiveness of GLP-1 RAs in preventing T2DM was low. Moreover, the only risk factor of T2DM that was considered for patients receiving SGLT2 inhibitors was CAD. However, we found a protective effect of SGLT2 in preventing T2DM in this high-risk group of patients. Finally, data concerning the combined action of SGT2 inhibitors and GLP-1 RAs were limited.

## 6 Conclusions

GLP-1 RAs may prevent T2DM in patients with obesity. Yet, the heterogeneity was large and the quality of evidence was low. Thus, the results should be interpreted with caution. Semaglutide was overall effective. Liraglutide might be effective in patients older than 40 years, at 3.0 mg daily, and after 55 weeks of intervention, while it seems that its effectiveness weakens after interruption. On the other hand, exenatide was not effective. Further research is needed to confirm our findings. Concerning safety, GLP-1 RAs were not associated with major adverse effects; however, they improved body and glycemic indices. The effectiveness of semaglutide at lower doses, liraglutide in men, and SGLT2 inhibitors in patients without CVD and the potential synergistic effect of GLP-1 RAs and SGLT2 inhibitors need further evaluation.

## Data Availability

The data analyzed in this study is subject to the following licenses/restrictions: The authors will provide the datasets upon reasonable request. Requests to access these datasets should be directed to gtsironikos@gmail.com.
