# Supplementary material for: Effectiveness of GLP-1 RAs and SGLT2 inhibitors in preventing T2DM in high-risk patients: an updated systematic review and meta-analysis
Source: Front Clin Diabetes Healthc. 2025 Dec 10;6:1694808. doi: 10.3389/fcdhc.2025.1694808 (PMC12727544; doi:10.3389/fcdhc.2025.1694808)
Supplement: Supplementary file 1 [file DataSheet1.docx]

**Table S1. Search strategy.**

| PubMed | ((diet OR nutrition) OR (exercise OR "physical activit*" OR workout) OR (lifestyle) OR (metformin) OR (GLP-1 RAs) OR (exenatide) OR (liraglutide) OR (albiglutide) OR (dulaglutide) OR (lixisenatide) OR (semaglutide) OR (tirzepatide) OR (SGLT2 inhibitors) OR (gliflozins) OR (dapagliflozin) OR (empagliflozin) OR (canagliflozin)) AND ((diabetes)) AND ("Clinical Trials as Topic"[Mesh] OR "randomized controlled trial"[pt] OR "controlled clinical trial"[pt] OR randomized[tiab] OR placebo[tiab] OR randomly[tiab] OR trial[tiab])  Filters applied: from 2000/1/1 – 2024/07/31 |
| --- | --- |
| CENTRAL | ((diet OR nutrition) OR (exercise OR “physical NEXT activit” OR workout) OR (lifestyle) OR (metformin) OR (GLP-1 RAs) OR (exenatide) OR (liraglutide) OR (albiglutide) OR (dulaglutide) OR (lixisenatide) OR (semaglutide) OR (tirzepatide) OR (SGLT2 inhibitors) OR (gliflozins) OR (dapagliflozin) OR (empagliflozin) OR (canagliflozin)) AND (diabetes)  with Cochrane Library publication date from Jan 2000 to Jul 2024, (Word variations have been searched) |
| Scopus | TITLE-ABS-KEY ( ( diet OR nutrition ) OR ( exercise OR "physical activit*" OR workout ) OR ( lifestyle ) OR ( metformin ) OR ( GLP-1 RAs ) OR ( exenatide ) OR ( liraglutide ) OR ( albiglutide ) OR ( dulaglutide ) OR ( lixisenatide ) OR ( semaglutide ) OR ( tirzepatide ) OR ( SGLT2 inhibitors ) OR ( gliflozins ) OR ( dapagliflozin ) OR ( empagliflozin ) OR ( canagliflozin ) AND ( diabetes ) ) AND ( LIMIT-TO ( DOCTYPE , "ar" ) ) AND ( LIMIT-TO ( LANGUAGE , "English" ) ) AND  ( LIMIT-TO ( PUBYEAR , 2024 ) OR LIMIT-TO ( PUBYEAR , 2023 ) OR LIMIT-TO ( PUBYEAR , 2022 ) OR LIMIT-TO ( PUBYEAR , 2021 ) OR LIMIT-TO ( PUBYEAR , 2020 ) OR LIMIT-TO ( PUBYEAR , 2019 ) OR LIMIT-TO ( PUBYEAR , 2018 ) OR LIMIT-TO ( PUBYEAR , 2017 ) OR LIMIT-TO ( PUBYEAR , 2016 ) OR LIMIT-TO ( PUBYEAR , 2015 ) OR LIMIT-TO ( PUBYEAR , 2014 ) OR LIMIT-TO ( PUBYEAR , 2013 ) OR LIMIT-TO ( PUBYEAR , 2012 ) OR LIMIT-TO ( PUBYEAR , 2011 ) OR LIMIT-TO ( PUBYEAR , 2010 ) OR LIMIT-TO ( PUBYEAR , 2009 ) OR LIMIT-TO ( PUBYEAR , 2008 ) OR LIMIT-TO ( PUBYEAR , 2007 ) OR LIMIT-TO ( PUBYEAR , 2006 ) OR LIMIT-TO ( PUBYEAR , 2005 ) OR LIMIT-TO ( PUBYEAR , 2004 ) OR LIMIT-TO ( PUBYEAR , 2003 ) OR LIMIT-TO ( PUBYEAR , 2002 ) OR LIMIT-TO ( PUBYEAR , 2001 ) OR LIMIT-TO ( PUBYEAR , 2000 ) ) |

**Table S2. Characteristics of Interventions and Comparators.**

| **First author’s name, publication year** | **Characteristics of compared arms** | | **Characteristics of Interventions** | | |
| --- | --- | --- | --- | --- | --- |
|  |  |  | **Dosage, description** | **Duration (wks)** | **Assessment of adherence** |
| **GLP-1 RAs Interventions** | | | | | |
| **Semaglutide-based Interventions** | | | | | |
| Wilding, 2022 [25] | Semaglutide plus lifestyle modification | Placebo plus Lifestyle modification | 2.4 mg sc per wk; starting dose 0.25 mg; titration every 4 wks until wk 16 (to doses of 0.5 mg, 1.0 mg, 1.7 mg, and 2.4 mg per wk). | 68 | NR |
|  |  |  | Counseling for 500 kcal deficit per day relative to total estimated energy expenditure at randomization. PA 150 min per wk. |  |  |
| Garvey, 2022 [26] | Semaglutide plus lifestyle modification | Placebo plus Lifestyle modification | 2.4 mg sc per wk ^†^; starting dose 0.25 mg; titration by 0.25 mg every 4 wks until wk 16. | 104 | NR |
|  |  |  | Counseling by a dietitian or similarly qualified healthcare professional to a reduced-calorie diet (500 kcal deficit a day relative to the energy expenditure estimated at randomization) and increased PA (150 min a wk). |  | In- person visits, telephone calls, diaries, apps. |
| Mu, 2024 [29] | Semaglutide plus lifestyle modification | Placebo plus Lifestyle modification | 2.4 mg sc per wk; starting dose 0.25 mg; titration every 4 wks until wk 16 (to doses of 0.5 mg, 1.0 mg, 1.7 mg, and finally 2.4 mg). | 44 | NR |
|  |  |  | Counseling by a dietitian or similarly qualified healthcare professional to a reduced-calorie diet (500 kcal deficit a day relative to the energy expenditure estimated at randomization) and increased PA (150 min a wk). |  | Clinic visits, telephone calls, diaries, apps. |
| Kahn, 2024 [30] | Semaglutide | Placebo | 2.4 mg sc per wk ^‡^; starting dose 0.6 mg; titration sequentially every 4 wks until wk 16. | 152 | NR |
| McGowan, 2024 [31] | Semaglutide plus lifestyle modification | Placebo plus Lifestyle modification | 2.4 mg sc per wk; starting dose 0.25 mg, titration every 4 wks until wk 16 (to doses of 0.5 mg, 1.0 mg, 1.7 mg, and 2.4 mg per wk). | 52 | NR |
|  |  |  | Counseling individually by a dietitian or similarly qualified healthcare professional on diet and PA for 52 wks. Counseling according to Sd clinical practice and country-specific guidelines for 28 wks. | 80 |  |
| **Liraglutide-based Interventions** | | | |  |  |
| Pi-Sunyer, 2015 [21] | Liraglutide plus lifestyle modification | Placebo plus Lifestyle modification | 3.0 mg sc once-daily; starting dose 0.6 mg; titration by 0.6 mg per wk for five wks. | 56 | NR |
|  |  |  | Counseling for reducing daily energy intake to 500 kcal below individualized energy requirements based on WHO estimates. Recommended macronutrient distribution 30% of energy from fat, 20% from protein, and 50% from carbohydrate. Counseling for increasing PA to at least 150 min per wk. | 68 | 3-day food diary, pedometers. |
| le Roux, 2017 [23] | Liraglutide plus lifestyle modification | Placebo plus Lifestyle modification | 3.0 mg sc once-daily; starting dose 0.6 mg; titration by 0.6 mg per wk for five wks. | 160 | NR |
|  |  |  | Counseling for reducing daily energy intake to 500 kcal below individualized energy requirements based on WHO estimates. Recommended macronutrient distribution 30% of energy from fat, 20% from protein, and 50% from carbohydrate. Counseling for increasing PA to at least 150 min per wk. | 172 | 3-day food diary, pedometers. |
| Svensson, 2019 [24] | Liraglutide | Placebo | 1.8 mg sc once-daily; starting dose 0.6 mg, titration during the initial two wks. | 16 | NR |
| Fogshsgaard, 2023 [27] | Liraglutide | Placebo | 1.8 mg sc once-daily ^§^; starting dose 0.6 mg; titration by 0.6 mg per wk. | 52 | NR |
| **Exenatide-based Intervention** | | | | | |
| Rosenstock, 2010 [20] | Exenatide plus lifestyle modification | Placebo plus Lifestyle modification | 10 mg sc equally divided twice daily; starting dose 5 mg for 4 wks. | 24 | NR |
|  |  |  | Structured program of diet and PA. |  |  |
| **SGLT2 Inhibitor Intervention** | | | | | |
| James, 2023 [28] | Dapagliflozin and Sd of care therapy ^¶^ | Placebo and Sd of care therapy ^¶^ | 10 mg orally once daily | 12 | NR |
| **GLP-1 RAs plus SGLT2 Inhibitor Intervention** | | | | | |
| Lundkvist, 2017 [22] | Dapagliflozin and Exenatide | Placebo | 10 mg orally once daily | 24 | Number of returned products, self-reported. |
|  |  |  | 2 mg sc per wk |  |  |

GLP-1 RAs, Glutagone-Like-Peptide-1 Receptor Agonists; RCTs; randomized control trials; mg, milligram; sc, subcutaneously; wk, week; NR, non-reported; kcal, kilocalorie; PA, physical activity; min, minute; apps, applications; Sd, standard; WHO, World Health Organization; SGLT2, Sodium-glucose coTrasnporter-2; mo, month. ^†^ Lower maintenance doses were permitted if participants were unable to tolerate 2.4 mg; ^‡^ Participants who did not tolerate dose escalation could be managed through extending dose escalation intervals, treatment pauses, or maintaining the dose at <2.4 mg per wk; ^§^ 1.2 mg for one participant due to side effects at 1.8 mg; ^¶^ In clinically stable patients hospitalized for myocardial infraction and with impaired left ventricle function without known diabetes or established heart failure.

**Table S3. T2DM assessment, efficacy of GLP-1 RAs and SGLT2 Inhibitors and T2DM diagnosis.**

| **First author’s name, publication year** | **Outcome of T2DM assessed as primary or secondary** | **Participants at risk for T2DM in analysis (I / C)** | **Events of T2DM; n (%) (I / C)** | **Diagnostic modalities** |
| --- | --- | --- | --- | --- |
| **GLP-1 RAs** | | | | |
| **Semaglutide** | | | | |
| Wilding, 2022 [25] | Secondary | 195 / 91 | 2 (1) / 3 (3.3) | HbA1c |
| Garvey, 2022 [26] | Secondary | 145 / 131 | 0 / 3 (2.3) | FPG, HbA1c |
| Mu, 2024 [29] | Secondary | 249 / 126 | 64 (25.7) / 32 (25.4) | FPG, HbA1c |
| Kahn, 2024 [30] | Primary | 8799 / 8795 | 306 (3.5) / 1059 (12) | HbA1c |
| McGowan, 2024 [31] | Secondary | 120 / 60 | 3 (2.5) / 5 (8.3) | FPG, HbA1c |
| **Liraglutide** | |  |  |  |
| Pi-Sunyer, 2015 [21] | Secondary | 2130 / 813 ^†^ | 4 (0.2) / 11 (1.3) ^‡^ | FPG, HbA1c, 2-h 75 g OGTT |
| le Roux, 2017 [23] | Primary | 1472 / 738 | 26 (1.7) / 46 (6.2) | FPG, HbA1c, 2-h 75 g OGTT |
| Svensson, 2019 [24] | Primary | 41 / 46 | 12 (26.2) / 6 (13) | FPG, HbA1c, random glucose measurement ^§^ |
| Fogshsgaard, 2023 [27] | Secondary | 49 / 55 | 4 (8.2) / 2 (3.6) | FPG, HbA1c, 4-h 75 g OGTT |
| **Exenatide** | | | | |
| Rosenstock, 2010 [20] | Secondary | 48 / 54 | 3 (6.2) / 2 (3.7) | FPG, 2-h 75 g OGTT |
| **SGLT2 Inhibitors** | | | | |
| James, 2023 [28] | Primary | 2019 / 1998 | 42 (2.1) / 78 (3.9) | HbA1c |
| **GLP-1 RAs plus SGLT2 Inhibitors** | | | | |
| Lundkvist, 2017 [22] | Secondary | 25 / 24 | 0 / 1 (4.2) | FPG, HbA1c, 2-h 75 g OGTT |

T2DM, Type-2 Diabetes Mellitus; GLP-1 RAs, Glutagone-Like-Peptide-1 Receptor Agonists; SGLT2, Sodium-glucose coTrasnporter-2; I, Intervention; C, Comparator; HbA1c, Hemoglobin A1c; FPG, fasting plasma glucose; h, hour; g, gram; OGTT, oral glucose tolerance test. ^†^ Participants at risk at week 56; ^‡^ Events of diabetes at week 56; ^§^ In patients with classic symptoms of hyperglycemia.

**Table S4. Subgroup and sensitivity analyses for GLP-1 Ras interventions.**

| **Subgroup Analyses** | | | | | | | | |
| --- | --- | --- | --- | --- | --- | --- | --- | --- |
| **Factor** | **Subgroup** | **No of studies** | **OR (95% CI)** | **P-value** | **Cochran’s Q statistic** | **Cochran’s Q statistic P-value** | **I^2^ (95% CI)** | **P-value test of difference** |
| Type of GLP1 RAs | Liraglutide | 4 | 0.64 (0.16, 2.53) | 0.53 | 21.88 | <0.0001 | 86% (38, 98%) | 0.34 |
|  | Semaglutide | 5 | 0.38 (0.16, 0.94) | 0.04 | 27.40 | <0.0001 | 85% (32, 97%) |  |
|  | Exenatide | 1 | 1.73 (0.16, 2.53) | 0.56 | N/A | N/A | N/A |  |
| **Semaglutide-based Subgroup Analyses** | | | | | | | | |
| Regions / Continents of multicenter RCTs | All continents | 1 | 0.26 (0.23, 0.30) | <0.00001 | N/A | N/A | N/A | <0.00001 |
|  | Europe, North America, Asia | 1 | 0.30 (0.05, 1.85) | 0.20 | N/A | N/A | N/A |  |
|  | North America, Europe | 2 | 0.24 (0.06, 0.90) | 0.03 | 0.24 | 0.63 | 0% (N/A) |  |
|  | Asia, South America | 1 | 1.02 (0.62, 1.66) | 0.95 | N/A | N/A | N/A |  |
| Post-intervention follow-up | Included | 2 | 0.29 (0.09, 0.91) | 0.03 | 0.00 | 0.95 | 0% (N/A) | 0.65 |
|  | Not included | 3 | 0.43 (0.13, 1.43) | 0.17 | 27.40 | <0.00001 | 93% (53, 99%) |  |
| Gender | Male more than female | 3 | 0.44 (0.15, 1.30) | 0.14 | 27.10 | <0.00001 | 93% (77, 99%) | 0.53 |
|  | Female more than male | 2 | 0.24 (0.05, 1.12) | 0.07 | 0.26 | 0.61 | 0% (N/A) |  |
| Mean age | More than 50 years | 2 | 0.26 (0.23, 0.30) | <0.00001 | 0.01 | 0.93 | 0% (N/A) | 0.16 |
|  | Less than 50 years | 3 | 0.59 (0.19, 1.79) | 0.35 | 3.32 | 0.19 | 40% (0, 98%) |  |
| CVD as main risk factor | Included^†^ | 1 | 0.26 (0.23, 0.30) | < 0.00001 | N/A | N/A | N/A | 0.11 |
|  | Not included | 4 | 0.50 (0.19, 1.29) | 0.15 | 5.45 | 0.14 | 45% (0, 91%) |  |
| Interventions’ duration | Less than 100 weeks | 3 | 0.58 (0.22, 1.50) | 0.26 | 3.92 | 0.14 | 49% (0, 98%) | 0.11 |
|  | More than 100 weeks | 2 | 0.26 (0.23, 0.30) | <0.00001 | 0.23 | 0.63 | 0% (N/A) |  |
| T2DM outcome assessment | Primary | 1 | 0.26 (0.23, 0.30) | <0.00001 | N/A | N/A | N/A | 0.19 |
|  | Secondary | 4 | 0.50 (0.19, 1.29) | 0.15 | 5.45 | 0.14 | 45% (0, 91%) |  |
| **Liraglutide-based Subgroup Analyses** | | | | | | | | |
| Regions / Continents of multicenter RCTs | All continents | 2 | 0.24 (0.14, 0.40) | <0.00001 | 1.14 | 0.29 | 12% (N/A) | <0.00001 |
|  | Europe (Denmark) | 2 | 2.64 (1.05, 6.65) | 0.04 | 0.02 | 0.88 | 0% (N/A) |  |
| Post-intervention follow-up | Included | 2 | 2.64 (1.05, 6.65) | 0.04 | 0.02 | 0.88 | 0% (N/A) | <0.00001 |
|  | Not included | 2 | 0.24 (0.14, 0.40) | <0.00001 | 1.14 | 0.29 | 12% (N/A) |  |
| Mean age | More than 40 years | 2 | 0.24 (0.14, 0.40) | <0.00001 | 1.14 | 0.29 | 12% (N/A) | 0.01 |
|  | Less than 40 years | 1 | 2.36 (0.41, 13.46) | 0.34 | N/A | N/A | N/A |  |
| Daily dosage | 1.8 mg | 2 | 2.64 (1.05, 6.65) | 0.04 | 0.02 | 0.88 | 0% (N/A) | <0.00001 |
|  | 3.0 mg | 2 | 0.24 (0.14, 0.40) | <0.00001 | 1.14 | 0.29 | 12% (N/A) |  |
| Interventions’ duration | Less than 55 weeks | 2 | 2.64 (1.05, 6.65) | 0.04 | 0.02 | 0.88 | 0% (N/A) | <0.00001 |
|  | More than 55 weeks | 2 | 0.24 (0.14, 0.40) | <0.00001 | 1.14 | 0.29 | 12% (N/A) |  |
| T2DM outcome assessment | Primary | 2 | 0.82 (0.08, 8.01) | 0.86 | 14.58 | 0.0001 | 93% | 0.81 |
|  | Secondary | 2 | 0.53 (0.03, 8.55) | 0.65 | 7.17 | 0.007 | 86% (0, 98%) |  |
| **Sensitivity Analyses** | | | | | | | | |
| **Semaglutide-based Sensitivity Analyses** | | | | | | | | |
| Effect of RCT with the largest sample size | Lower sample size | 4 | 0.50 (0.19, 1.29) | 0.15 | 5.45 | 0.14 | 45% (0, 91%) | |
| Effect of RCTs with post-intervention follow-up | Without post-intervention follow-up | 3 | 0.43 (0.13, 1.43) | 0.17 | 27.40 | <0.00001 | 93% 93% (53, 99%) | |
| Effect of RCTs with drop-out rate more than 5% | With drop-out rate less than 5% | 2 | 0.51 (0.13, 1.90) | 0.31 | 27.10 | <0.00001 | 96% (17, 98%) | |
| **Liraglutide-based Sensitivity Analyses** | | | | | | | | |
| Effect of RCT with the largest sample size | Lower sample size | 3 | 1.10 (0.18, 6.72) | 0.92 | 18.33 | 0.0001 | 89% (7, 99%) | |
| Effect of RCTs with post-intervention follow-up | Without post-intervention follow-up | 2 | 0.24 (0.14, 0.40) | <0.00001 | 1.14 | 0.29 | 12% (N/A) | |
| Effect of RCTs with drop-out rate more than 10% | With drop-out rate less than 10% | 3 | 0.36 (0.11, 1.16) | 0.09 | 7.26 | 0.03 | 72% (0, 97%) | |

GLP-1 Ras, Glucagon-Like Peptide-1 Receptor Agonists; No, numbers; CI, confidence interval; N/A, not applicable; RCT, randomized controlled trials; CVD, cardiovascular disease; T2DM, Type-2 Diabetes Mellitus; GDM, gestational diabetes mellitus. ^†^ Additionally to overweight/obesity

**Table S5. Description of changes in weight, BMI, HbA1c, FPG, and 2-h OGTT.**

| **First author’s name, publication year** | **Sample size (I / C)** | **Weight (kg) mean difference vs placebo (std deviation)** | **BMI (kg/m²) mean difference vs placebo (std deviation)** | **HbA1c (%) mean difference vs placebo (std deviation)** | **FPG (mmol/L) mean difference vs placebo (std deviation)** | **2-h OGTT (mmol/L) mean difference vs placebo (std deviation)** |
| --- | --- | --- | --- | --- | --- | --- |
| **GLP-1 RAs** | | | | | | |
| **Semaglutide** | | | | | | |
| Wilding, 2022 [25] | 195 / 91 | –6.6 / 0.1 (0.7 / 3.1) | –2.6 / –0.1 (0.1 / 0.2) | –0.1 / 0 (0 / 0.2) | N/R | N/R |
| Garvey, 2022 [26] | 145 / 131 | –16.1 / –3.2 (1 / 1.2) | –5.9 / –1.6 (0.4 / 0.6) | –0.4 / –0.1 (0.03 / 0.03) | –0.4 / 0.1 (0.05 / 0.06) | N/R |
| Mu, 2024 [29] | 249 / 126 | –11.5 / –3.4 (0.5 / 0.7) | –4.1 / –1.2 (0.2 / 0.3) | –0.8 / –0.1 (0.1 / 0.1) | –1.0 / –0.1 (0.1 / 0.1) | N/R |
| Kahn, 2024 [30] | 8799 / 8795 | ‒9.5 / ‒0.85 (17.58 / 17.58) | NR | ‒0.27 / 0.05 (0.33 / 0.33) | NR | NR |
| McGowan, 2024 [31] | 120 / 60 | –8.7 / –1.2 (8.4 / 6.0) | NR | –0.1 / 0 (0.3 / 0.2) | –0.3 / –0.1 (0.7 / 0.8) | N/R |
| **Liraglutide** | | | | | | |
| Pi-Sunyer, 2015 [21] | 2130 / 813 | ‒8.4 / ‒2.8 (7.3 / 6.5) | NR | −0.30 / −0.06 (0.28 / 0.30) | ‒0.4 / 0 (0.6 / 0.6) | N/R |
| le Roux, 2017 [23] | 1472 / 738 | –6.5 / –2.0 (8.1 / 7.3) | –2.4 / –0.7 (2.9 / 2.6) | –0.35 / –0.14 (0.32 / 0.34) | –0.37 / 0.05 (0.68 / 0.62) | –1.6 / –0.2 (2.1 / 2.2) |
| Svensson, 2019 [24] | 33 / 40 | ‒2.3 / ‒0.1 (1.5 / 1.4) | ‒0.7 / 0.2 (0.4 / 0.2) | 2.3 / 2.2 (2.2 / 2.2) | 1.02 / 0.98 (1.02 / 1.02) | N/R |
| Fogshsgaard, 2023 [27] | 49 / 55 | ‒4.9 / ‒1.0 (1.69 / 1.6) | NR | ‒2.6 / 2.2 (2.3 / 2.2) | ‒0.27 / ‒0.03 (0.84 / 0.45) | N/R |
| **Exenatide** | | | | | | |
| Rosenstock, 2010 [20] | 48 / 54 | ‒5.1 / ‒1.6 (0.5 / 0.5) | NR | NR | NR | N/R |
| **SGLT2 Inhibitors** | | | | | | |
| James, 2023 [28] | 2019 / 1998 | ‒1.41 / 0.24 (0.34, 0.35) | N/R | N/R | N/R | N/R |
| **GLP-1 RAs plus SGLT2 Inhibitors** | | | | | | |
| Lundkvist, 2017 [22] | 25 / 24 | −4.48 / −0.35 (1.6 / 0.35) | N/R | −0.36 / −0.15 (0.08 / 0.09) | −0.41 / 0.25 (0.2 / 0.21) | −1.57 / −0.08 (0.79 / 0.85) |

BMI, body mass index; HbA1c, hemoglobin A1c; FPG, fasting plasma glucose; h, hour; OGTT, oral glucose tolerance test; No, number; I, intervention; C, control; kg, kilogram; std, standard; m, meter; mmol, millimole; L, liter; N/R, non-report.

**Table S6. Secondary outcomes in GLP-1 RAs RCTs.**

| **Outcomes** | **No of studies** | **Sample size (I / C)** | **Mean difference vs placebo (95%CI)** | **P-value** | **P-value test of difference** |
| --- | --- | --- | --- | --- | --- |
| Weight (kg) | 10 | 13.240 / 10,903 | ‒6.35 (‒8.63, ‒4.07) | < 0.00001 |  |
| - Weight and Semaglutide | 5 | 9508 / 9203 | ‒8.82 (‒11.47, ‒6.16) | < 0.00001 | 0.0004 |
| - Weight and Liraglutide | 4 | 3684 / 1646 | ‒4.06 (‒5.47, ‒2.64) | < 0.00001 |  |
| - Weight and Exenatide | 1 | 48 / 54 | ‒3.50 (‒3.69, ‒3.31) | < 0.00001 |  |
| BMI (kg/m²) | 5 | 2094 / 1126 | ‒2.46 (‒3.17, ‒1.76) | < 0.00001 |  |
| - BMI and Semaglutide | 3 | 589 / 348 | ‒3.23 (‒3.94, ‒2.52) | < 0.00001 | 0.0003 |
| - BMI and Liraglutide | 2 | 1505 / 778 | ‒1.29 (‒2.08, ‒0.51) | 0.001 |  |
| HbA1c (%) | 9 | 13,192 / 10,849 | ‒0.37 (‒0.48, ‒0.27) | < 0.00001 |  |
| - HbA1c and Semaglutide | 5 | 9508 / 9203 | ‒0.36 (‒0.50, ‒0.22) | < 0.00001 | 0.66 |
| - HbA1c and Liraglutide | 4 | 3684 / 1646 | ‒0.41 (‒0.60, ‒0.22) | < 0.0001 |  |
| FPG (mmol/L) | 7 | 4198 / 1963 | ‒0.42 (‒0.62, ‒0.21) | < 0.0001 |  |
| - FPG and Semaglutide | 3 | 514 / 317 | ‒0.55 (‒0.88, ‒0.22) | 0.001 | 0.35 |
| - FPG and Liraglutide | 4 | 3684 / 1646 | ‒0.39 (‒0.46, ‒0.33) | < 0.00001 |  |
| Major adverse events | 5 | 4116 / 1868 | 1.01 (0.76, 1.35) | 0.95 |  |
| - Major adverse events and Semaglutide | 3 | 514 / 317 | 0.73 (0.44, 1.22) | 0.23 | 0.13 |
| - Major adverse events and Liraglutide | 2 | 3602 / 1551 | 1.17 (0.83, 1.66) | 0.37 |  |

GLP-1 RAs, Glutagone-Like-Peptide-1 Receptor Agonists; No, number; I, intervention; C, control; CI, confidence interval; kg, kilogram; m, meter; mmol, millimole; L, liter; OR, odds ratio.

**Table S7. Safety of GLP-1 RAs and SGLT2 inhibitors interventions.**

| **First author’s name, publication year** | **Adverse events, side effects as reported; n, patients (%) (I / C)** |
| --- | --- |
| **GLP-1 RAs** | |
| **Semaglutide** | |
| Wilding, 2022 [25] | NR |
| Garvey, 2022 [26] | Any adverse event: 146 (96.1) / 136 (89.5) |
|  | Serious adverse events: 12 (7.9) / 18 (11.8) |
|  | Adverse events leading to trial product discontinuation: 9 (5.9) / 7 (4.6) |
|  | Gastrointestinal disorders leading to trial product discontinuation: 6 (3.9) / 1 (0.7) |
|  | Fatal events: 1 (0.7) / 0 |
|  | Adverse events reported in at least 10% of participants: Nausea 81 (53.3) / 33 (21.7), Diarrhea 53 (34.9) / 36 (23.7), Constipation 47 (30.9) / 17 (11.2), Vomiting 46 (30.3) / 7 (4.6), Nasopharyngitis 24 (15.8) / 23 (15.1), Abdominal pain upper 22 (14.5) / 10 (6.6), Abdominal pain 20 (13.2) / 4 (2.6), Dyspepsia 20 (13.2) / 7 (4.6), Flatulence 20 (13.2) / 10 (6.6), Gastroenteritis 20 (13.2) / 4 (2.6), Influenza 20 (13.2) / 16 (10.5), Upper respiratory tract infection 20 (13.2) / 23 (15.1), Decreased appetite 17 (11.2) / 6 (3.9), Eructation 17 (11.2) / 1 (0.7), Headache 16 (10.5) / 16 (10.5), Back pain 15 (9.9) / 19 (12.5) |
|  | Safety areas of interest: Gastrointestinal disorders 125 (82.2) / 82 (53.9). Gallbladder-related disorders 4 (2.6) / 2 (1.3), Hepatobiliary disorders 4 (2.6) / 2 (1.3), Cholelithiasis 3 (2.0) / 2 (1.3), Hepatic disorders 3 (2.0) / 3 (2.0), Acute pancreatitis 0 / 0 , Cardiovascular disorders 17 (11.2) / 32 (21.1), Allergic reactions 23 (15.1) / 8 (5.3), Injection-site reactions 10 (6.6) / 15 (9.9), Malignant neoplasms 2 (1.3) / 4 (2.6), Psychiatric disorders 26 (17.1) / 25 (16.4), Acute renal failure 0 / 0, Hypoglycemia 4 (2.6) / 0, Rare events 0 / 1 (0.7%), Overdose 0 / 1 (0.7%), COVID-19 16 (10.5) / 8 (5.3) |
| Mu, 2024 [29] | Any adverse events: 231 (93) / 108 (86) |
|  | Serious adverse events: 13 (5) / 8 (6) |
|  | Adverse events leading to trial product discontinuation: 7 (3) / 2 (2) |
|  | Gastrointestinal disorders leading to trial product discontinuation: 3 (1) / 0 |
|  | Fatal events: 0 / 0 |
|  | Treatment-emergent adverse events reported in at least 5% of participants: Diarrhoea 65 (26) / 13 (10), Nausea 60 (24) / 9 (7), Upper respiratory tract infection 53 (21) /26 (21), Decreased appetite 43 (17) / 5 (4), Constipation 29 (12) / 7 (6), Abdominal distension 25 (10) / 1 (1), Vomiting 21 (8) /0, Abdominal pain upper 16 (6)/ 4 (3), Arthralgia 15 (6) / 3 (2), COVID-19 15 (6) /5 (4), Dyspepsia 14 (6) / 0, Eructation 14 (6) / 0, Abdominal pain 13 (5) / 3 (2), Dizziness 13 (5) / 6 (5) |
|  | Adverse events of special interest: Gastrointestinal adverse events 168 (67) / 45 (36), Cardiovascular disorders 33 (13) / 8 (6), Allergic reaction 20 (8) / 7 (6), Psychiatric disorder adverse events 13 (5) / 9 (7), Neoplasms 12 (5) / 7 (6), Hepatic events 8 (3) / 9 (7), Acute gallbladder disease 4 (2) / 3 (2), Rare events 3 (1) / 1 (1), Acute renal failure 2 (1) / 0, Injection site reaction 2 (1%) / 0, Malignant neoplasms 1 (<1) / 1 (1) , Acute pancreatitis 0 / 0, Medication error 0 / 0, Misuse or abuse of trial product 0 / 0 |
| Kahn, 2024 [30] | NR |
| McGowan, 2024 [31] | Serious adverse events total: 12 (9) / 6 (9); Serious adverse events leading to study drug discontinuation 4 (3) / 0, Serious adverse events with fatal outcome 2 (1) / 0, Treatment-emergent serious adverse events reported in at least 5% of participants 0 / 0 |
|  | Serious adverse events by system organ class: Gastrointestinal disorders 3 (2) / 0, Infections and infestations 3 (2) / 2 (3), Neoplasms benign, malignant, and unspecified (including cysts and polyps) 3 (2) / 0, Cardiac disorders 2 (1) / 3 (4), Injury, poisoning, and procedural complications 2 (1) / 0, Reproductive system and breast disorders 2 (1) / 0, Eye disorders 1 (1) / 0, General disorders and administration site conditions 1 (1) / 1 (1), Hepatobiliary disorders 1 (1) / 0, Musculoskeletal and tissue disorders 0 / 1 (1), Product issues 0 / 1 (1), Vascular disorders 0 / 1 (1) |
|  | Adverse events of special interest: COVID-19 (on treatment) 49 (36) / 24 (35), Cardiovascular disorders (in trial) ^1^ 4 (3) / 3 (4), Neoplasms (in trial) ^†^ 4 (3) / 0, Gastrointestinal disorders (on treatment) ^†^ 3 (2) / 0, Acute pancreatitis (on treatment) 2 (1) / 0, Malignant neoplasms (in trial) ^†^ 2 (1) / 0, Medication errors (on treatment) 2 (1) / 1 (1), Acute gallbladder disease (on treatment) ^1^ 1 (1) / 0, Misuse and abuse (on treatment) 0 / 0 |
| **Liraglutide** | |
| Pi-Sunyer, 2015 [21] | Adverse events in ≥ 5% of patients: Nausea 997 (40.2) / 183 (14.7), Diarrhea 518 (20.9) / 115 (9.3), Constipation 495 (20.0) / 108 (8.7), Vomiting 404 (16.3) / 51 (4.1), Dyspepsia 236 (9.5) / 39 (3.1), Upper abdominal pain 141 (5.7) / 43 (3.5), Abdominal pain 130 (5.2) / 43 (3.5), Nasopharyngitis 427 (17.2) / 234 (18.8), Upper respiratory tract infection 213 (8.6) / 122 (9.8), Sinusitis 128 (5.2) / 73 (5.9), Influenza 144 (5.8) / 66 (5.3), Headache 327 (13.2) / 154 (12.4), Dizziness 167 (6.7) / 60 (4.8), Decreased appetite 267 (10.8) / 38 (3.1), Back pain 171 (6.9) / 105 (8.5), Arthralgia 125 (5.0) / 71 (5.7), Fatigue 185 (7.5) / 65 (5.2), Injection-site hematoma 142 (5.7) / 93 (7.5) |
|  | Serious adverse events in ≥ 0.2% of patients: Cholelithiasis 20 (0.8) / 5 (0.4), Cholecystitis acute 12 (0.5) / 0, Osteoarthritis 6 (0.2) / 0, Intervertebral disc protrusion 5 (0.2) / 1 (0.1), Pancreatitis acute 4 (0.2) / 0 ^‡^, Cholecystitis 4 (0.2) / 0, Breast cancer 4 (0.2) /1 (0.1%), Back pain 2 (0.1) / 2 (0.2), Uterine leiomyoma 1 (<0.1) / 2 (0.2), Cellulitis 1 (<0.1) / 3 (0.2), Gastroesophageal reflux disease 0 / 2 (0.2), Bronchitis 0 / 2 (0.2), Bladder prolapse 0 / 2 (0.2), Chest pain 0 / 3 (0.2) |
| le Roux, 2017 [23] | Adverse events in ≥ 5% of patients: 1322 (88) / 579 (78); Gastrointestinal disorders: Nausea 614 (41.0) / 125 (17.0), Diarrhea 379 (25.0) / 107 (14.0), Constipation 331 (22.0) / 85 (11.0), Vomiting 295 (20.0) / 40 (5.0), Dyspepsia 154 (10.0) / 35 (5.0), Abdominal pain 114 (8.0) / 38 (5.0), Upper abdominal pain 112 (8.0) / 39 (5.0), Gastro-esophageal reflux disease 98 (7) / 18 (2), Eructation 85 (6) / 4 (<1), Flatulence 81 (5) / 20 (3) |
|  | General disorders and administration site conditions: Fatigue 152 (10) / 57 (8), Injection site haematoma 91 (6) / 60 (8), Oedema peripheral 53 (4) / 47 (6) |
|  | Infections and infestations: Nasopharyngitis 396 (26) / 209 (28), Upper respiratory tract infection 235 (16) / 119 (16), Influenza 181 (12) / 79 (11), Gastroenteritis 142 (9) / 46 (6), Sinusitis 128 (9) / 65 (9), Urinary tract infection 121 (8) / 43 (6), Bronchitis 114 (8) / 62 (8) |
|  | Investigations Lipase increased 146 (10) / 23 (3) |
|  | Metabolism and nutrition disorders: Decreased appetite 164 (11) / 26 (4) |
|  | Musculoskeletal and connective tissue disorders: Back pain 200 (13) / 120 (16), Arthralgia 184 (12) / 97 (13), Pain in extremity 108 (7) / 54 (7) |
|  | Nervous system disorders: Headache 270 (18) / 122 (16), Dizziness 146 (10) / 54 (7) |
|  | Respiratory, thoracic, and mediastinal disorders: Cough 111 (7) / 59 (8), Oropharyngeal pain 74 (5) / 44 (6) |
|  | Vascular disorders: Hypertension 75 (5) / 47 (6) |
|  | Serious adverse events in ≥ 0.4% of individuals: Cholelithiasis 20 (1) / 6 (1), Cholecystitis acute 9 (1) / 1 (<1), Cholecystitis 6 (<1) / 0, Osteoarthritis 12 (1) / 5 (1), Intervertebral disc protrusion 6 (<1) / 1 (<1), Back pain 4 (<1) / 3 (<1), Fall 0 / 4 (1), Cellulitis 3 (<1) / 3 (<1), Obesity 1 (<1) / 3 (<1) |
| Svensson, 2019 [24] | NR |
| Fogshsgaard, 2023 [27] | NR |
| **Exenatide** | |
| Rosenstock, 2010 [20] | Nausea 18 (25) / 3 (4), diarrhea 10 (14) / 2 (3) ^§^ |
| **SGLT2 Inhibitors** | |
| James, 2023 [28] | No increase in serious adverse events related to adverse reactions that could potentially be associated with SGLT2 inhibitors, such as ketoacidosis, hypovolemia, hypotension, amputations, or genital infections. |
| **GLP-1 RAs plus SGLT2 Inhibitors** | |
| Lundkvist, 2017 [22] | Participants with at least one adverse event: Any adverse event 25 (100.0) / 25 (100.0) |
|  | Any serious adverse event: 1 (4.0) / 1 (4.0) |
|  | Treatment-related adverse events: 4 (16.0) / 3 (12.0) |
|  | Adverse events leading to study discontinuation: 2 (8.0) / 3 (12.0) |
|  | Deaths: 0 / 0 |
|  | Adverse events of special interest: Urinary tract infections 2 (8.0) / 1 (4.0), Acute pyelonephritis 1 (4.0) / 0 (0.0), Fungal urinary tract infection 1 (4.0) / 0 |
|  | Genital infections: Vaginal infection 1 (4.0%) / 0 |
|  | Volume reduction: Hypotension 0 / 1 (4.0) |
|  | Renal impairment/failure: 0 / 0 |
|  | Gastrointestinal symptoms: Nausea 7 (28.0) / 3 (12.0), Diarrhea 3 (12.0), 3 (12.0), Abdominal distension 3 (12.0) / 2 (8.0), Vomiting 3 (12.0) / 1 (4.0), Gastroesophageal reflux 3 (12.0) / 1 (4.0), Constipation 2 (8.0) / 1 (4.0), Dyspepsia 2 (8.0) / 0, Abdominal pain 1 (4.0) / 0 |
|  | Injection-site disorders: Injection-site mass 7 (28.0) / 5 (20.0), Injection-site pruritus 7 (28.0) / 2 (8.0), Injection-site erythema 3 (12.0) / 1 (4.0), Injection-site nodule 2 (8.0) / 1 (4.0), Injection-site swelling 0 / 2 (8.0), Injection-site pain 1 (4.0) / 0, Injection-site cyst 1 (4.0) / 0, Injection-site rash 0 / 1 (4.0) |
|  | Appetite changes: Decreased appetite 8 (32.0) / 3 (12.0), Increased appetite 1 (4.0) / 0, Hunger 1 (4.0) 3 / (12.0) |

GLP-1 RAs, Glutagone-Like-Peptide-1 Receptor Agonists; SGLT2, Sodium-glucose coTrasnporter-2; I, Intervention; C, Comparator; NR, non-reported; COVID-19, Coronavirus disease 2019. ^†^ Only serious adverse events were considered; ^‡^ Pancreatitis acute reported as serious by the investigator but classified as mild according to revised Atlanta classification of acute pancreatitis; ^§^ Majority of symptoms mild-to-moderate.

**Table S8. Quality of reporting for eligible studies.**

| **First author, publication year** | **R Bias arising from the randomisation process judgment (low, high, some concerns)** | **D Bias due to deviations from intended interventions judgment (low, high, some concerns)** | **Mi Bias due to missing outcome data judgment (low, high, some concerns)** | **Me Bias in measurement of the outcome judgment (low, high, some concerns)** | **S Bias in selection of the reported result judgment (low, high, some concerns)** | **O Overall risk of bias judgment (low, high, some concerns)** |
| --- | --- | --- | --- | --- | --- | --- |
| **GLP-1 RAs** | | | | | | |
| **Semaglutide-based RCTs** | | | | | | |
| Wilding, 2022 [25] | some concerns | low | low | some concerns | low | some concerns |
| Garvey, 2022 [26] | some concerns | low | low | some concerns | low | some concerns |
| Mu, 2024 [29] | low | low | low | low | low | low |
| Kahn, 2024 [30] | some concerns | low | low | some concerns | low | some concerns |
| McGowan, 2024 [31] | some concerns | low | low | low | low | some concerns |
| **Liraglutide-based RCTs** | | | | | | |
| Pi-Sunyer, 2015 [21] | some concerns | low | high | low | low | high |
| le Roux, 2017 [23] | low | low | high | low | low | high |
| Svensson, 2019 [24] | some concerns | low | low | some concerns | low | some concerns |
| Fogshsgaard, 2023 [27] | low | low | low | low | low | low |
| **Exenatide-based RCT** | | | | | | |
| Rosenstock, 2010 [20] | some concerns | high | high | some concerns | some concerns | high |
| **SGLT2 Inhibitors** | | | | | | |
| James, 2023 [28] | low | low | low | low | low | low |
| **GLP-1 RAs plus SGLT2 Inhibitors** | | | | | | |
| Lundkvist, 2017 [22] | high | low | high | low | low | high |

GLP-1 RAs, Glutagone-Like-Peptide-1 Receptor Agonists; RCTs; randomized control trials; SGLT2, Sodium-glucose coTrasnporter-2

**Fig. S1. Subgroup analysis based on type of GLP-1 RAs.**

**
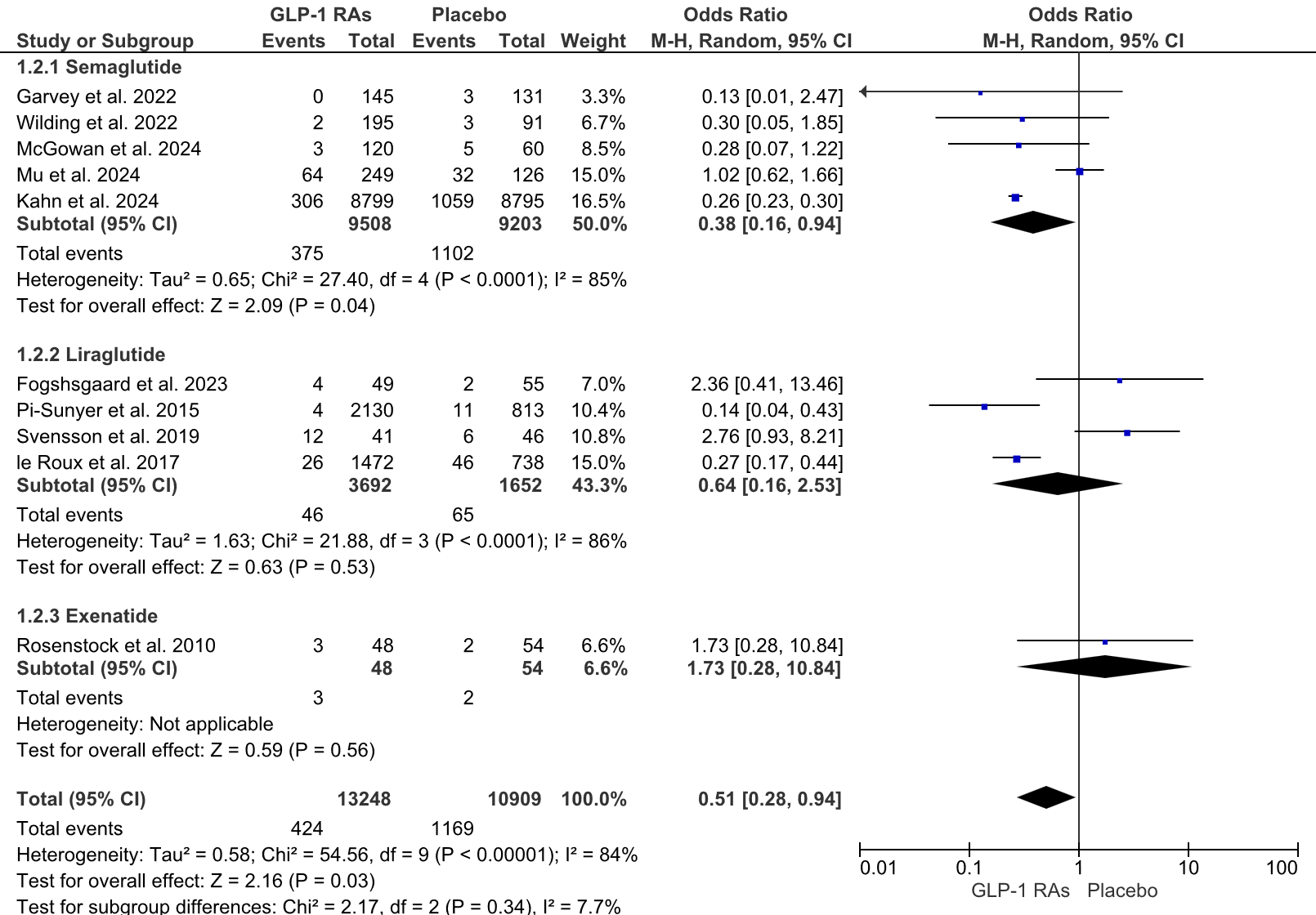
**

Events, number of participants with Type-2 Diabetes Mellitus; Total, number of participants at risk for Type-2 Diabetes Mellitus; Blue dots, weight of studies; Black blocks, 95% confidence interval of studies; Diamond, estimate with 95% confidence interval

**Fig. S2. Subgroup analysis in semaglutide based on similar countries.**

**
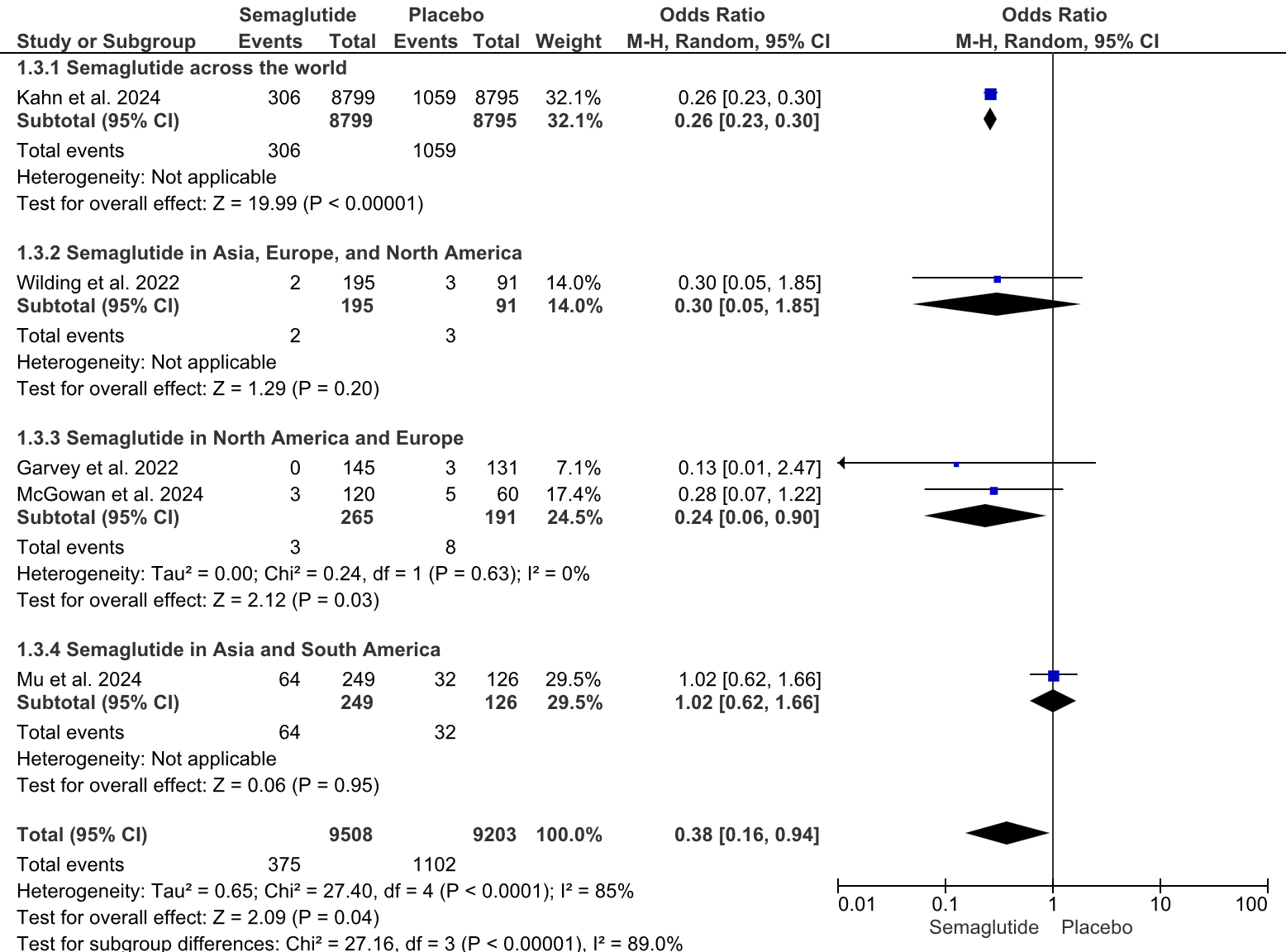
**

Events, number of participants with Type-2 Diabetes Mellitus; Total, number of participants at risk for Type-2 Diabetes Mellitus; Blue dots, weight of studies; Black blocks, 95% confidence interval of studies; Diamond, estimate with 95% confidence interval

**Fig. S3. Subgroup analysis in semaglutide based on post-intervention follow-up.**

**
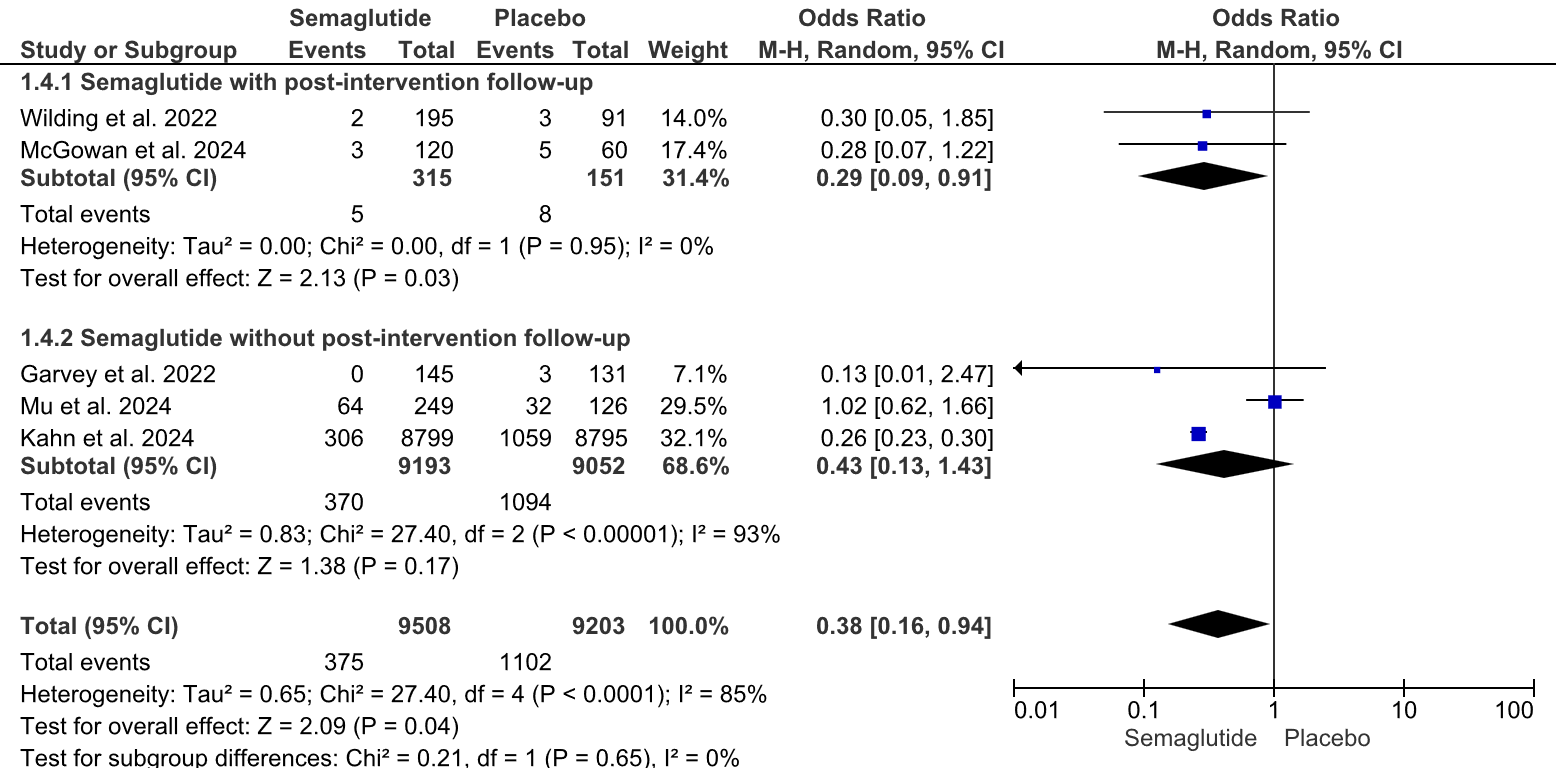
**

Events, number of participants with Type-2 Diabetes Mellitus; Total, number of participants at risk for Type-2 Diabetes Mellitus; Blue dots, weight of studies; Black blocks, 95% confidence interval of studies; Diamond, estimate with 95% confidence interval

**Fig. S4. Subgroup analysis in semaglutide based on participants’ gender.**

**
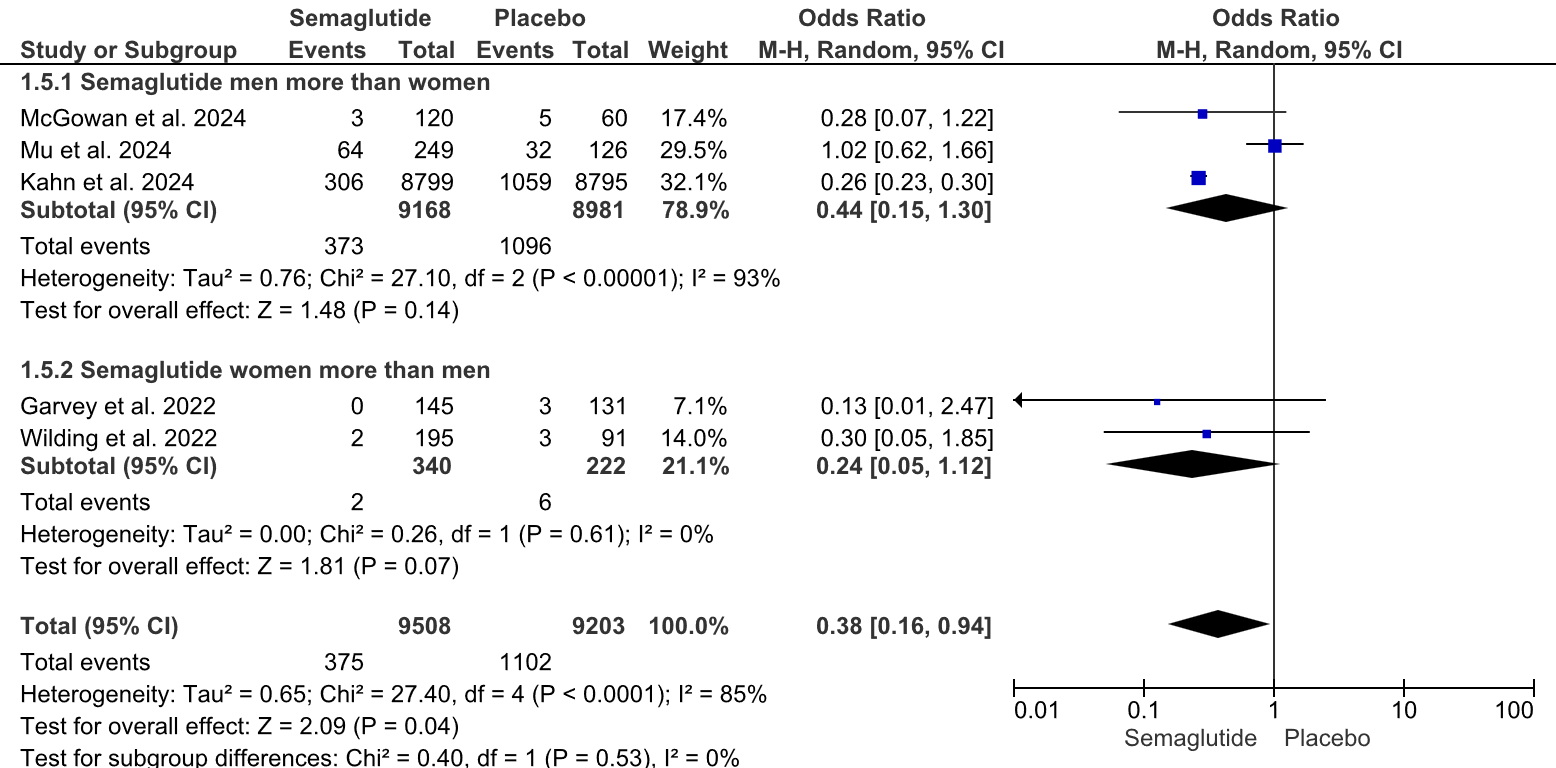
**

Events, number of participants with Type-2 Diabetes Mellitus; Total, number of participants at risk for Type-2 Diabetes Mellitus; Blue dots, weight of studies; Black blocks, 95% confidence interval of studies; Diamond, estimate with 95% confidence interval

**Fig. S5. Subgroup analysis in semaglutide based on participants’ mean age.**

**
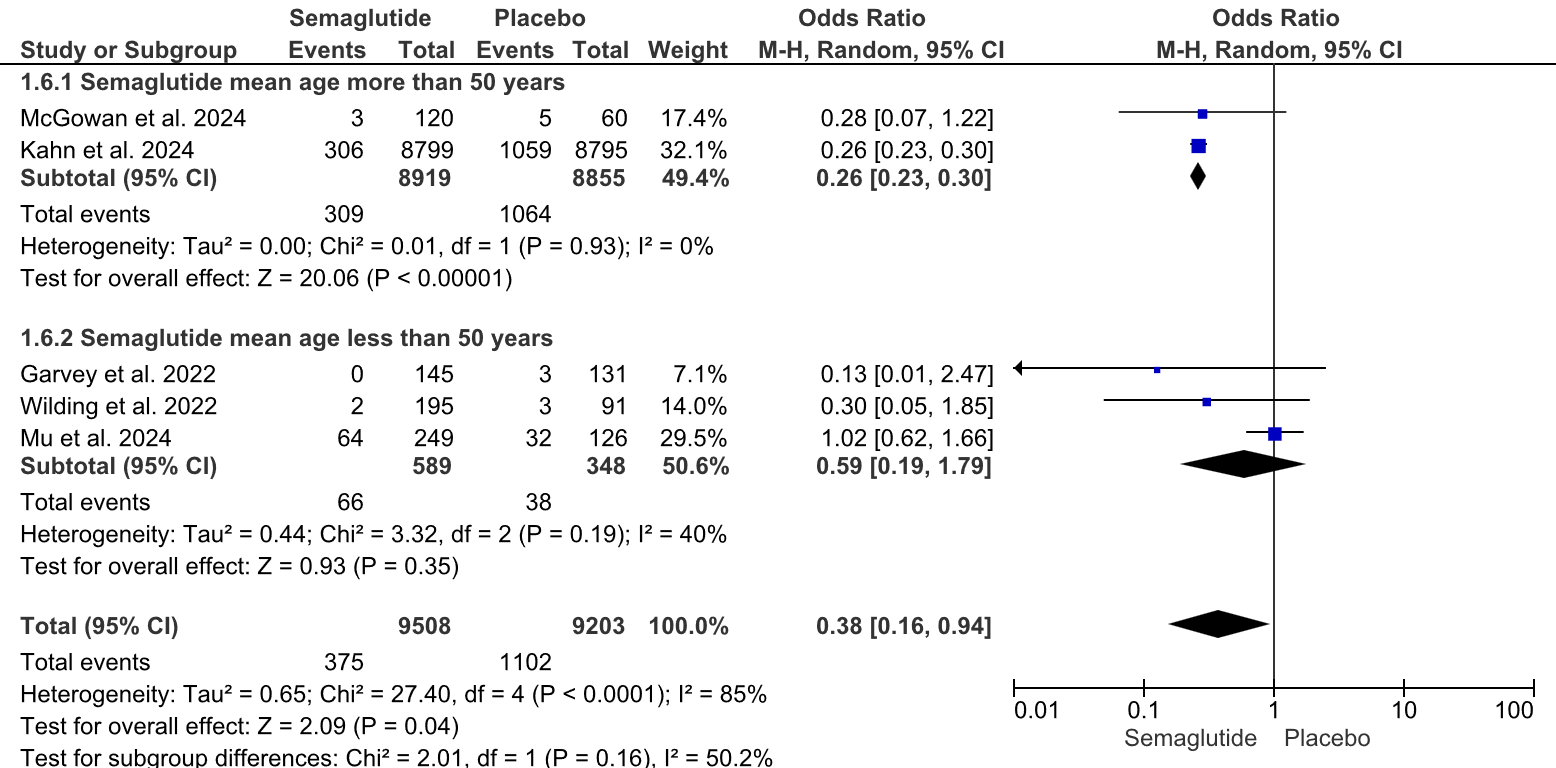
**

Events, number of participants with Type-2 Diabetes Mellitus; Total, number of participants at risk for Type-2 Diabetes Mellitus; Blue dots, weight of studies; Black blocks, 95% confidence interval of studies; Diamond, estimate with 95% confidence interval

**Fig. S6. Subgroup analysis in semaglutide based on CVD as additional diabetes’ risk factor.**

**
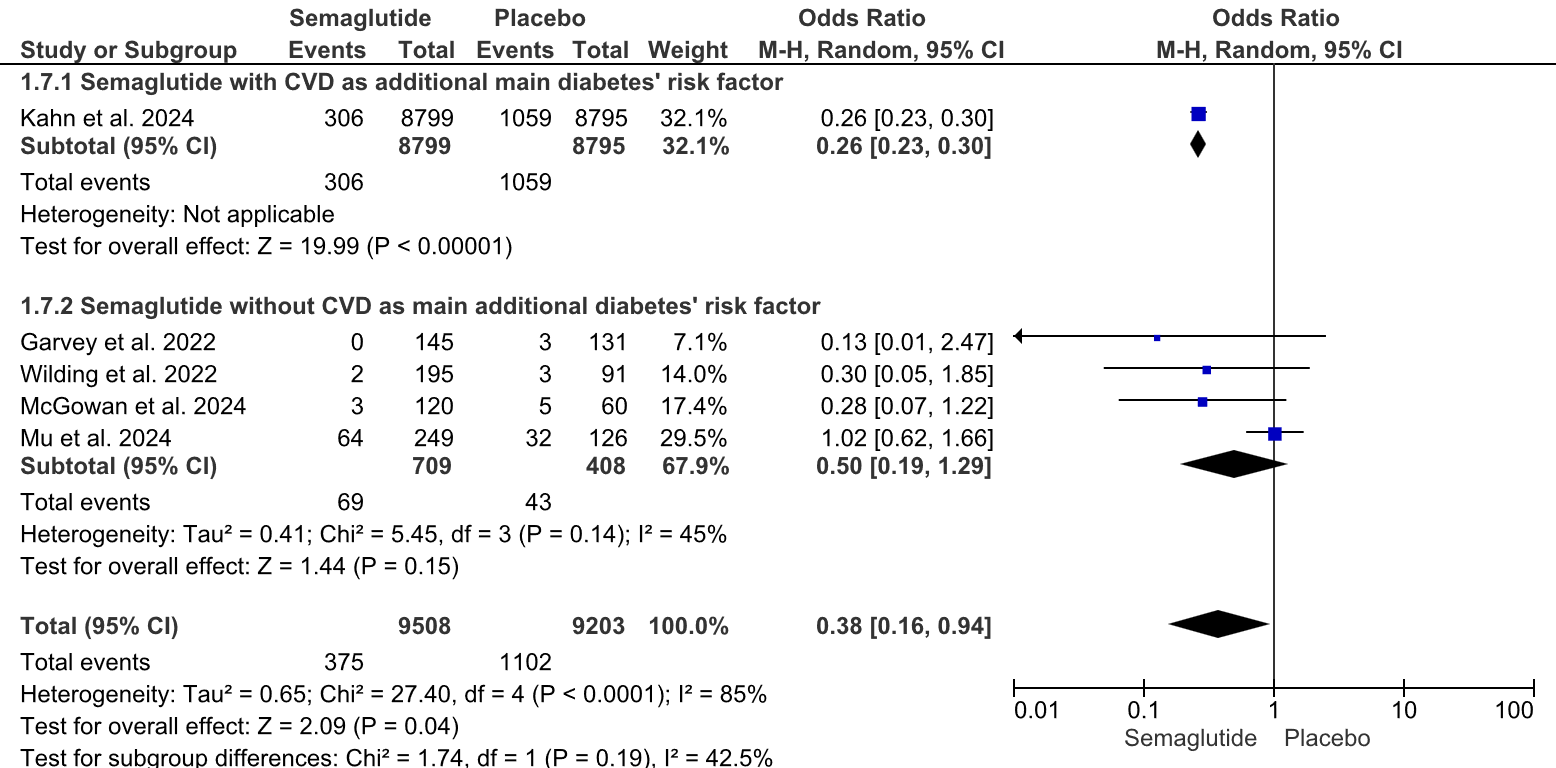
**

Events, number of participants with Type-2 Diabetes Mellitus; Total, number of participants at risk for Type-2 Diabetes Mellitus; Blue dots, weight of studies; Black blocks, 95% confidence interval of studies; Diamond, estimate with 95% confidence interval

**Fig. S7.** **Subgroup analysis in semaglutide based on intervention’s duration.**

**
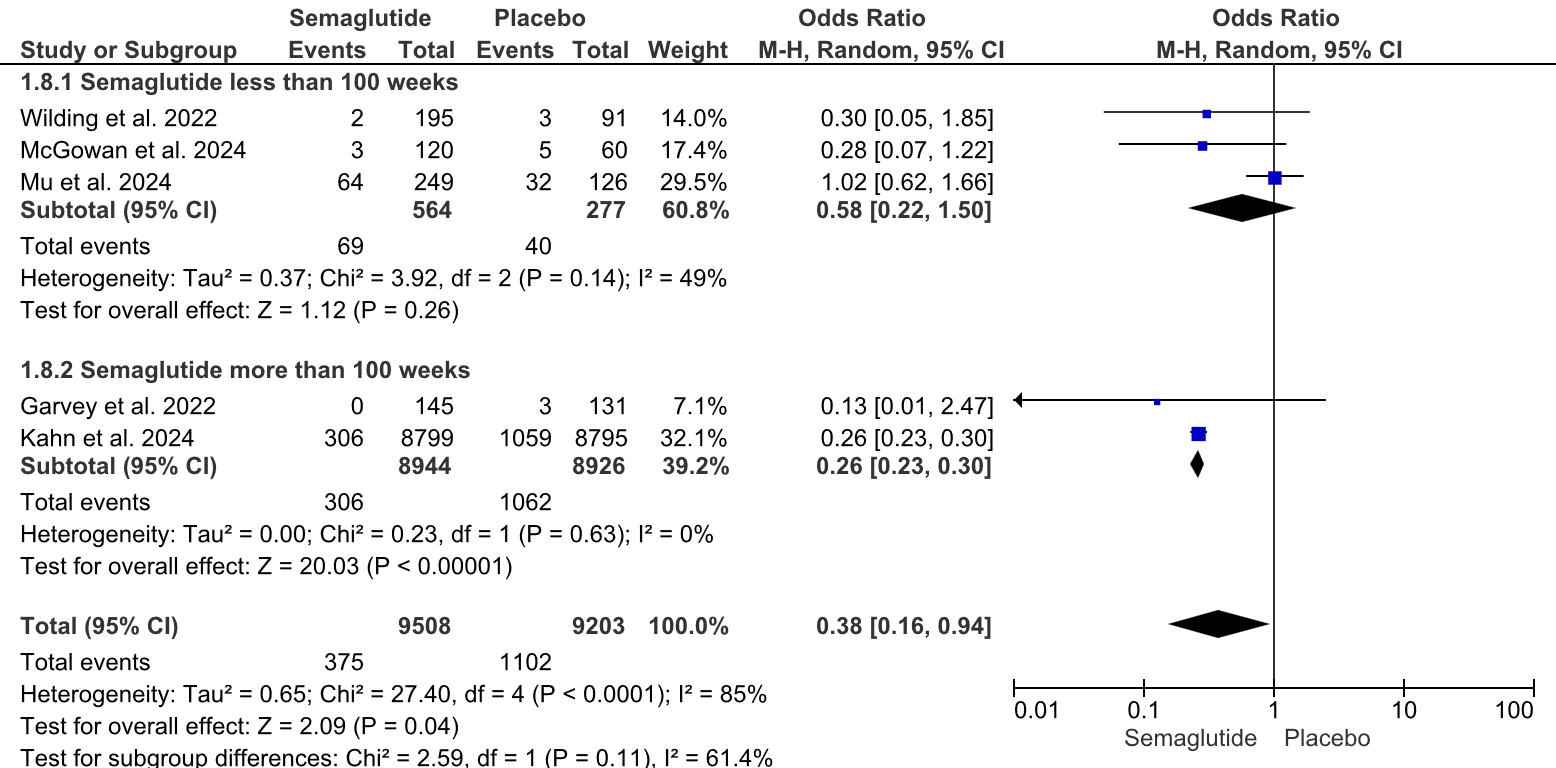
**

Events, number of participants with Type-2 Diabetes Mellitus; Total, number of participants at risk for Type-2 Diabetes Mellitus; Blue dots, weight of studies; Black blocks, 95% confidence interval of studies; Diamond, estimate with 95% confidence interval

**Fig. S8.** **Subgroup analysis in semaglutide based on diabetes’ outcome assessment.**

**
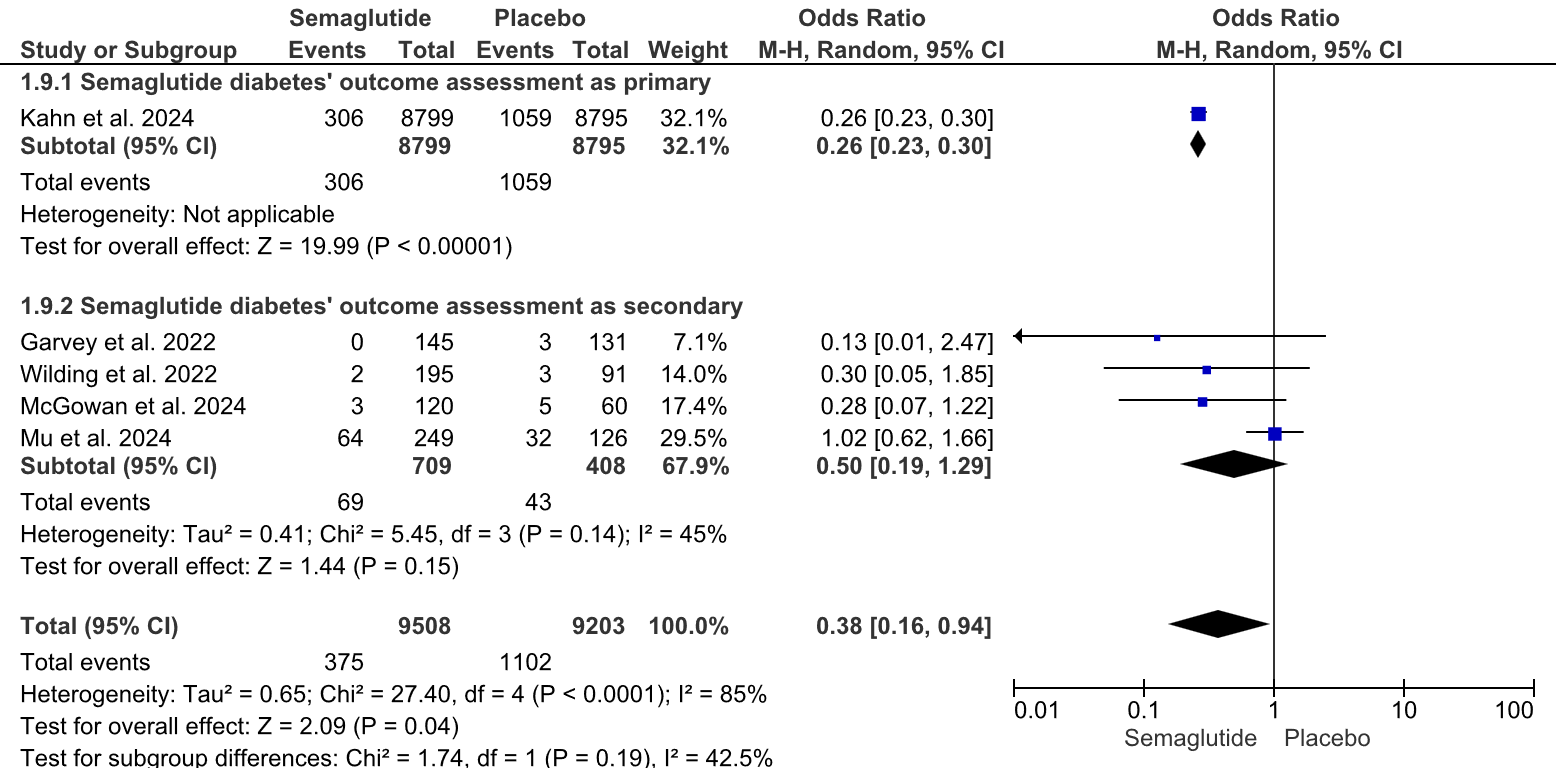
**

Events, number of participants with Type-2 Diabetes Mellitus; Total, number of participants at risk for Type-2 Diabetes Mellitus; Blue dots, weight of studies; Black blocks, 95% confidence interval of studies; Diamond, estimate with 95% confidence interval

**Fig. S9. Sensitivity analysis in semaglutide for the study with largest sample size.**


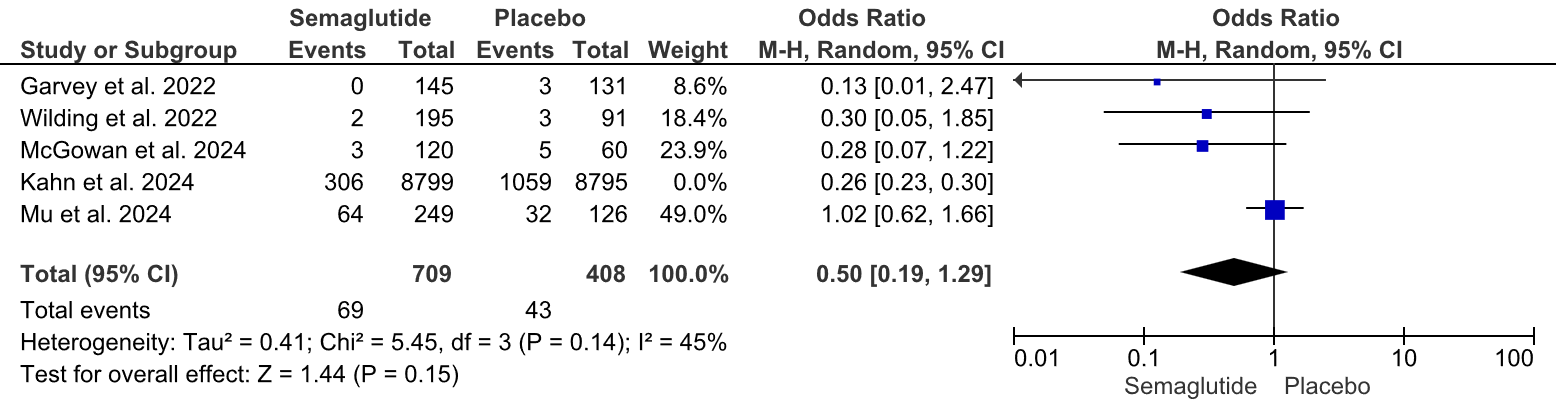
Events, number of participants with Type-2 Diabetes Mellitus; Total, number of participants at risk for Type-2 Diabetes Mellitus; Blue dots, weight of studies; Black blocks, 95% confidence interval of studies; Diamond, estimate with 95% confidence interval

**Fig. S10. Sensitivity analysis in semaglutide for the studies with post-intervention.**


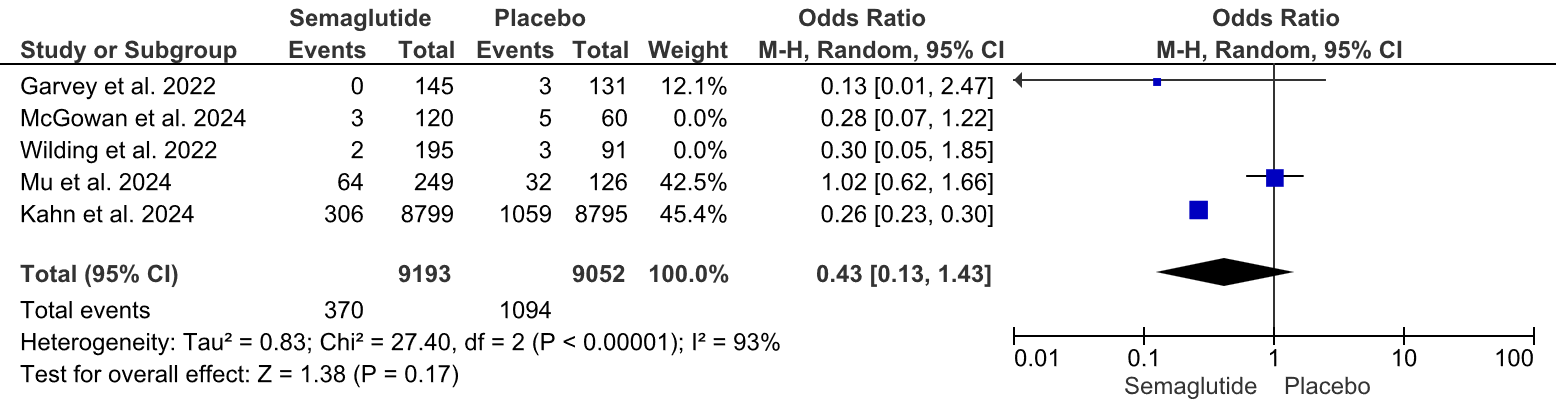
Events, number of participants with Type-2 Diabetes Mellitus; Total, number of participants at risk for Type-2 Diabetes Mellitus; Blue dots, weight of studies; Black blocks, 95% confidence interval of studies; Diamond, estimate with 95% confidence interval

**Fig. S11. Sensitivity analysis in semaglutide for the studies with drop-out rate more than 5%.**


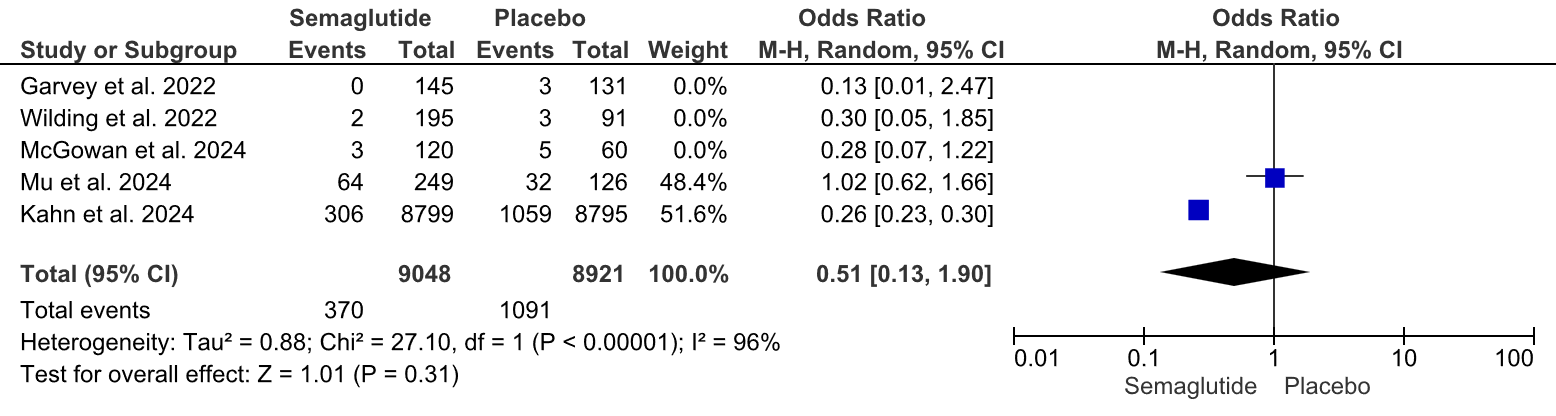
Events, number of participants with Type-2 Diabetes Mellitus; Total, number of participants at risk for Type-2 Diabetes Mellitus; Blue dots, weight of studies; Black blocks, 95% confidence interval of studies; Diamond, estimate with 95% confidence interval

**Fig. S12. Subgroup analysis in liraglutide based on similar countries.**

**
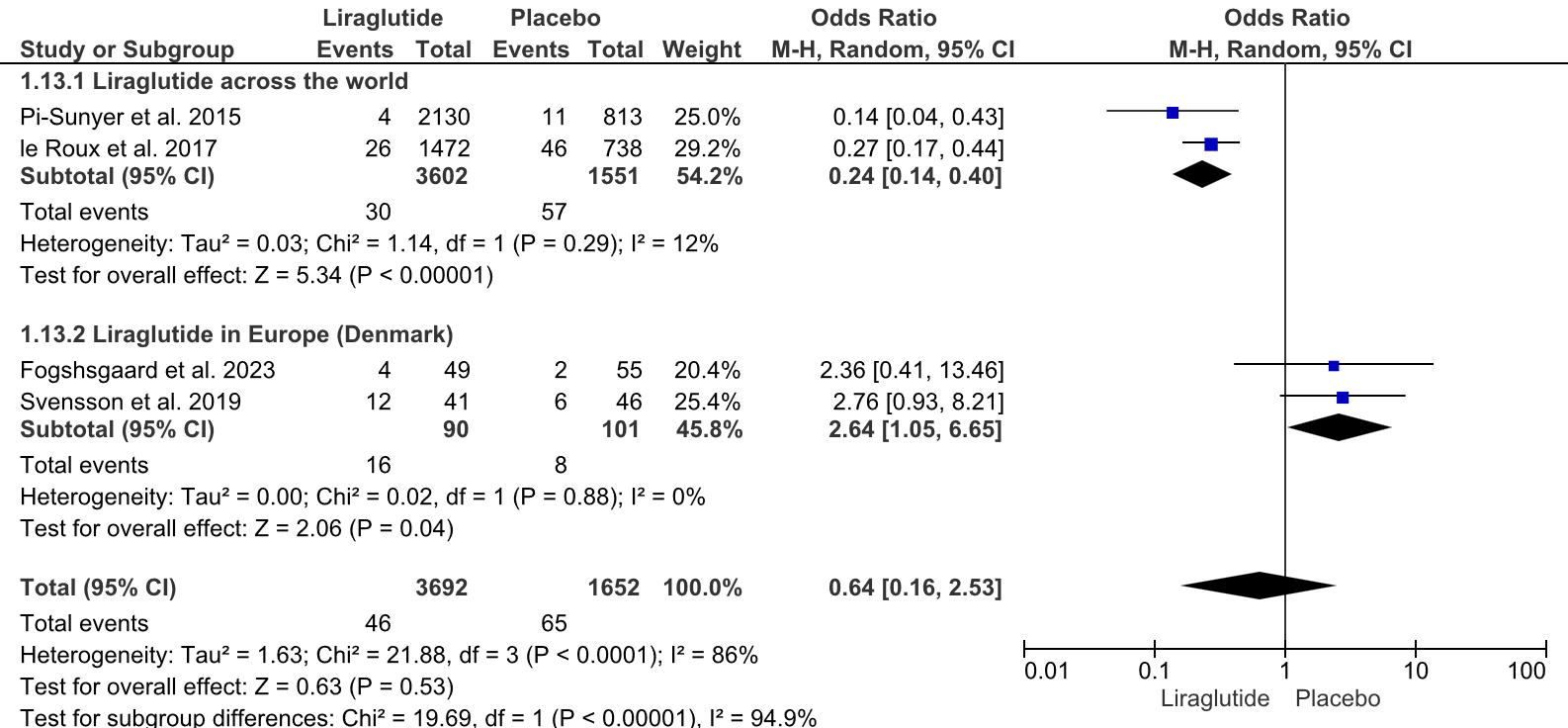
**

Events, number of participants with Type-2 Diabetes Mellitus; Total, number of participants at risk for Type-2 Diabetes Mellitus; Blue dots, weight of studies; Black blocks, 95% confidence interval of studies; Diamond, estimate with 95% confidence interval

**Fig. S13. Subgroup analysis in liraglutide based on post-intervention follow-up.**

**
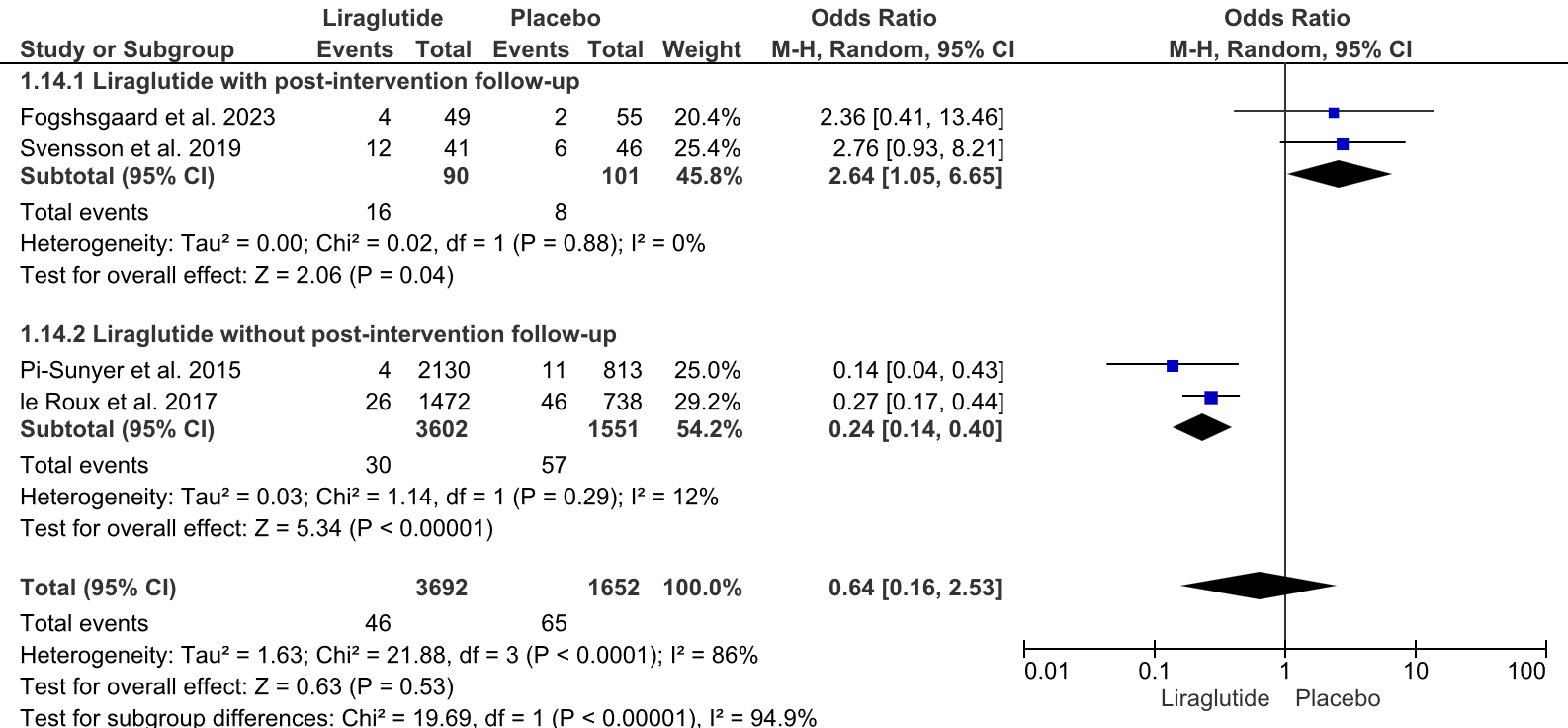
**

Events, number of participants with Type-2 Diabetes Mellitus; Total, number of participants at risk for Type-2 Diabetes Mellitus; Blue dots, weight of studies; Black blocks, 95% confidence interval of studies; Diamond, estimate with 95% confidence interval

**Fig. S14. Subgroup analysis in liraglutide based on participants’ mean age.**

**
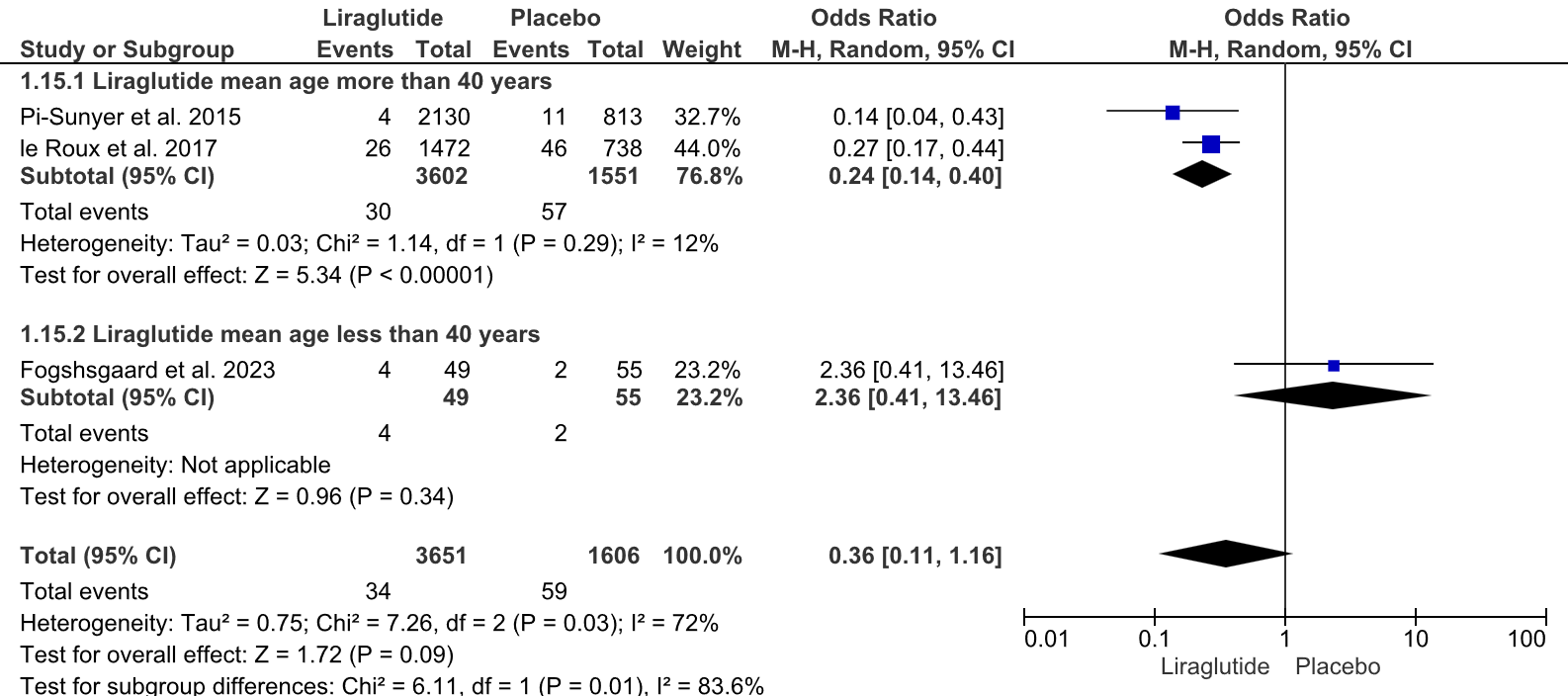
**

Events, number of participants with Type-2 Diabetes Mellitus; Total, number of participants at risk for Type-2 Diabetes Mellitus; Blue dots, weight of studies; Black blocks, 95% confidence interval of studies; Diamond, estimate with 95% confidence interval

**Fig. S15. Subgroup analysis in liraglutide based on daily dosage.**

**
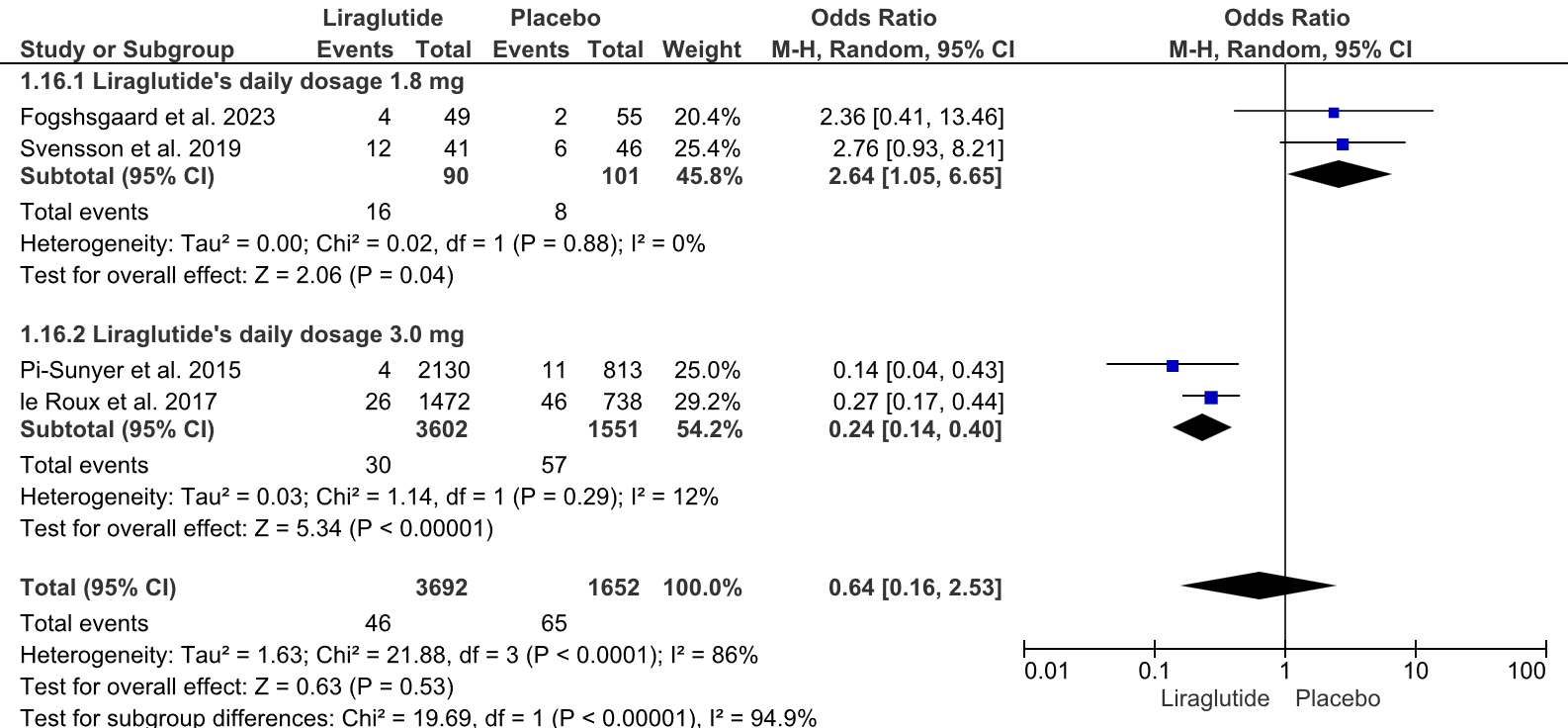
**

Events, number of participants with Type-2 Diabetes Mellitus; Total, number of participants at risk for Type-2 Diabetes Mellitus; Blue dots, weight of studies; Black blocks, 95% confidence interval of studies; Diamond, estimate with 95% confidence interval

**Fig. S16. Subgroup analysis in liraglutide based on intervention’s duration.**

**
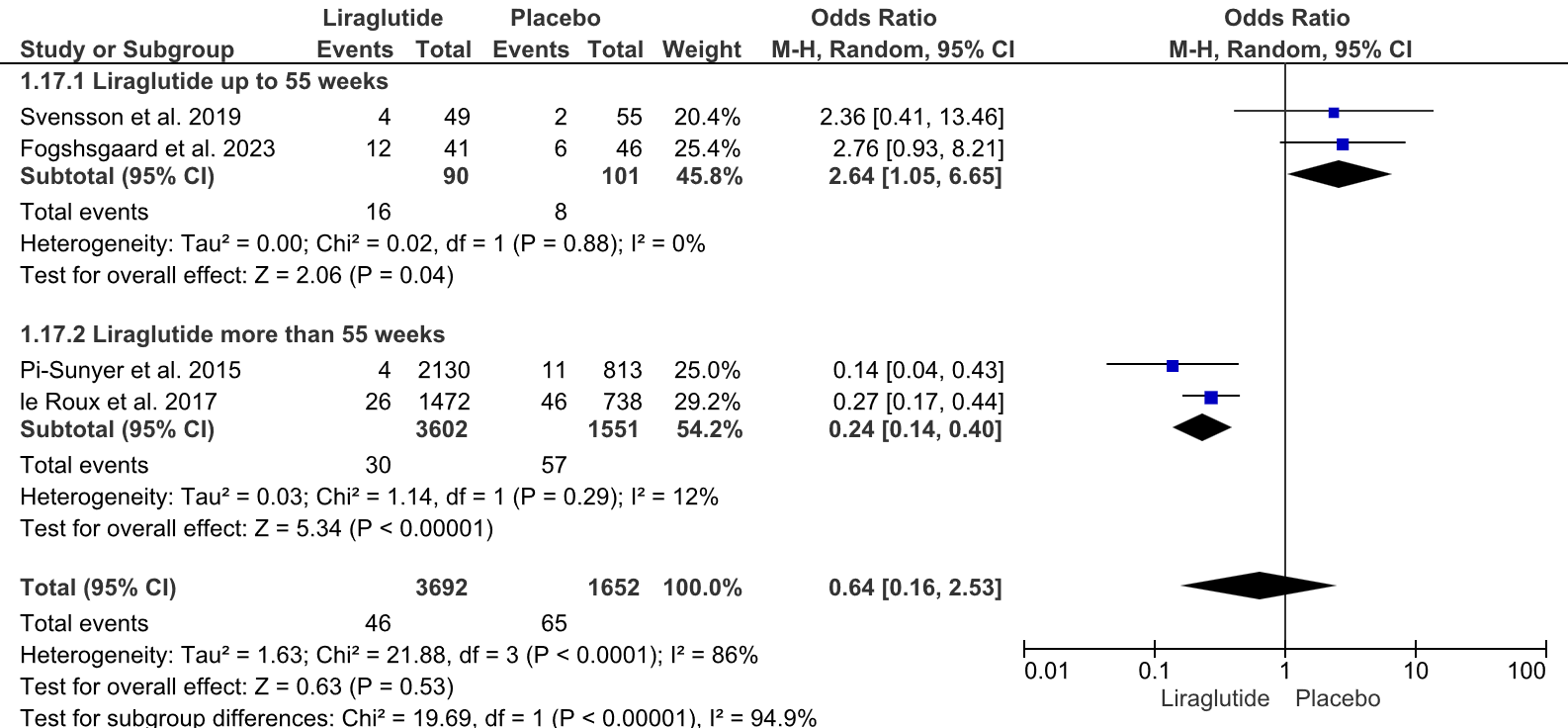
**

Events, number of participants with Type-2 Diabetes Mellitus; Total, number of participants at risk for Type-2 Diabetes Mellitus; Blue dots, weight of studies; Black blocks, 95% confidence interval of studies; Diamond, estimate with 95% confidence interval

**Fig. S17. Subgroup analysis in liraglutide based on diabetes’ outcome assessment.**

**
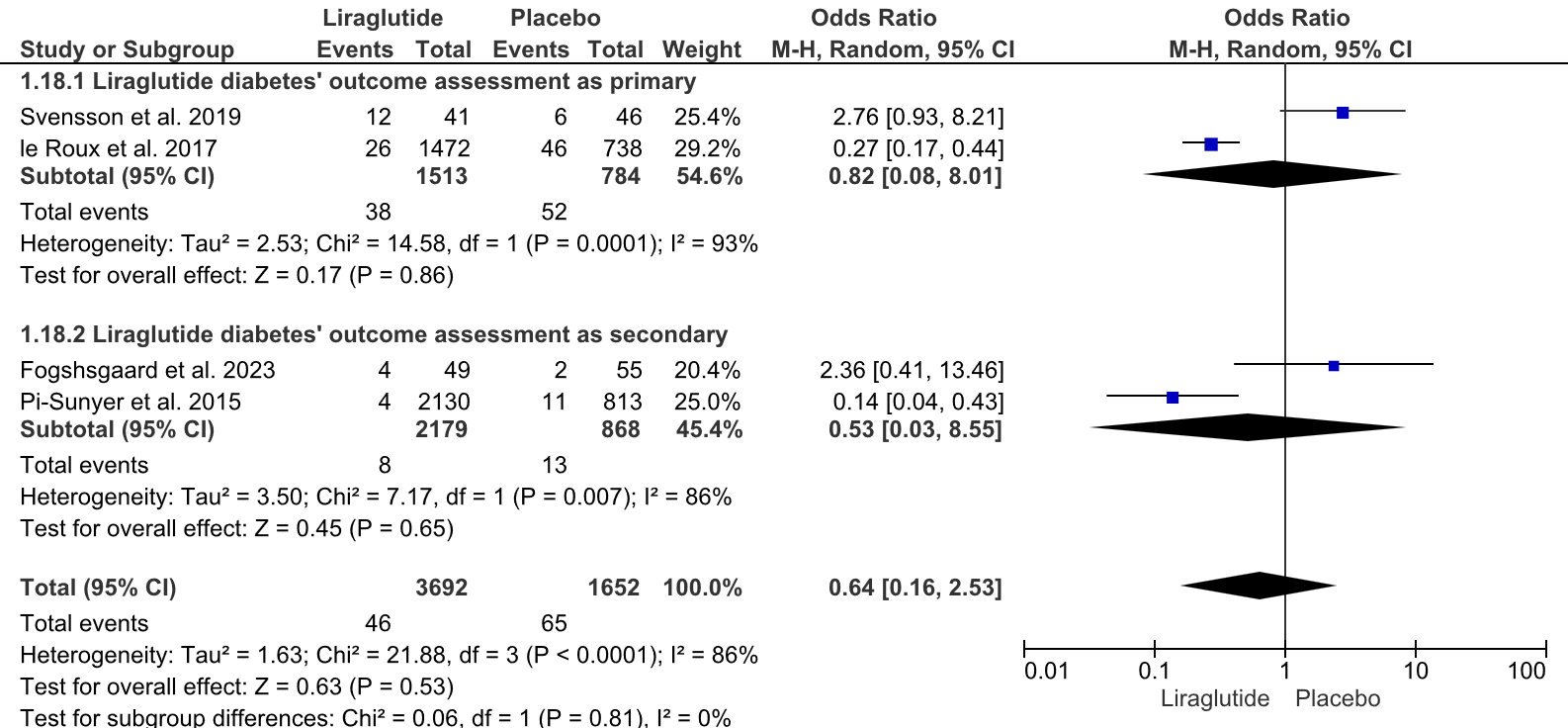
**

Events, number of participants with Type-2 Diabetes Mellitus; Total, number of participants at risk for Type-2 Diabetes Mellitus; Blue dots, weight of studies; Black blocks, 95% confidence interval of studies; Diamond, estimate with 95% confidence interval

**Fig. S18. Sensitivity analysis in liraglutide for the study with largest sample size.**


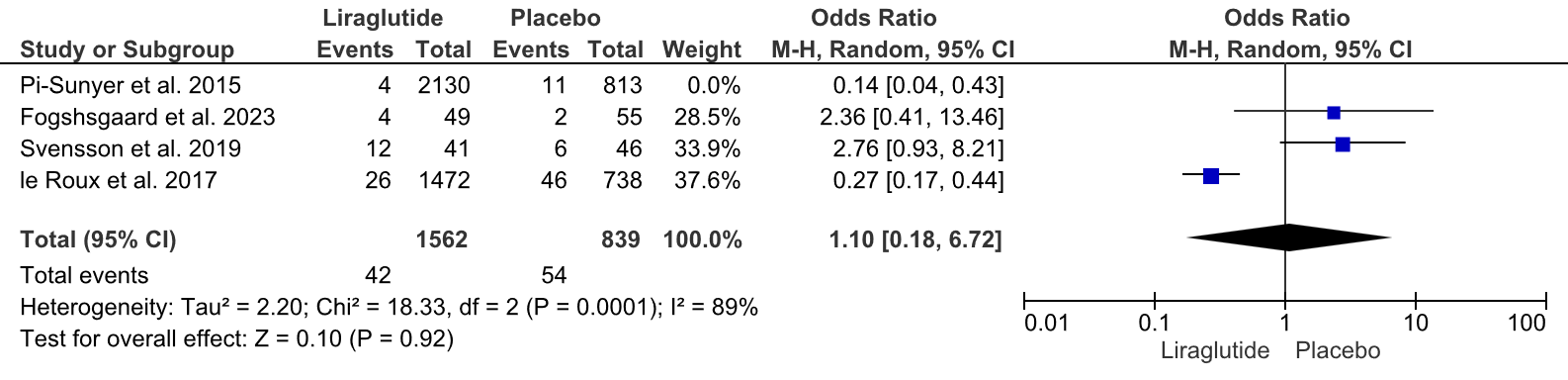
Events, number of participants with Type-2 Diabetes Mellitus; Total, number of participants at risk for Type-2 Diabetes Mellitus; Blue dots, weight of studies; Black blocks, 95% confidence interval of studies; Diamond, estimate with 95% confidence interval

**Fig. S19. Sensitivity analysis in liraglutide for the studies with post-intervention.**


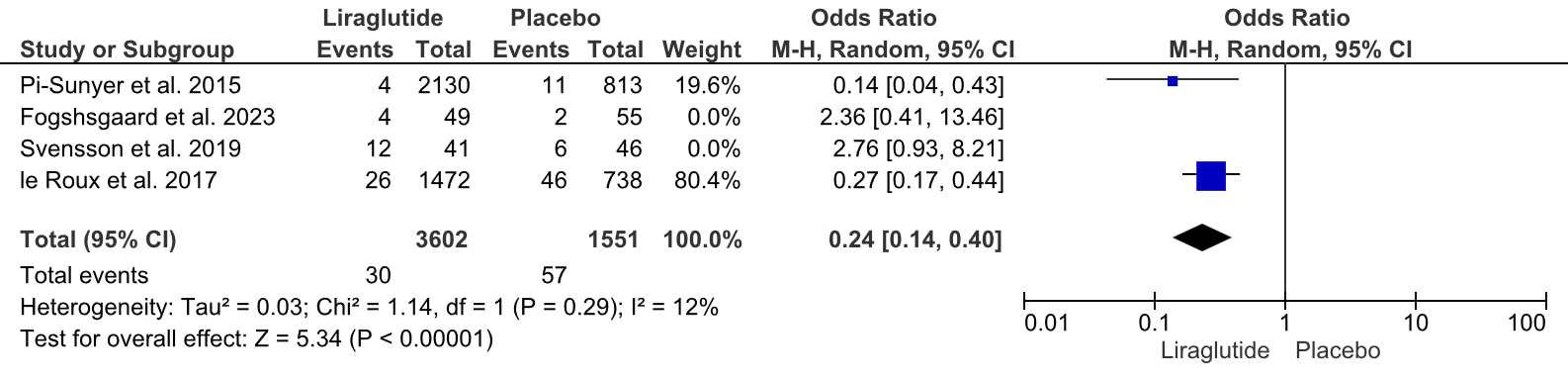
Events, number of participants with Type-2 Diabetes Mellitus; Total, number of participants at risk for Type-2 Diabetes Mellitus; Blue dots, weight of studies; Black blocks, 95% confidence interval of studies; Diamond, estimate with 95% confidence interval

**Fig. S20. Sensitivity analysis in liraglutide for the study with drop-out rate more than 10%.**


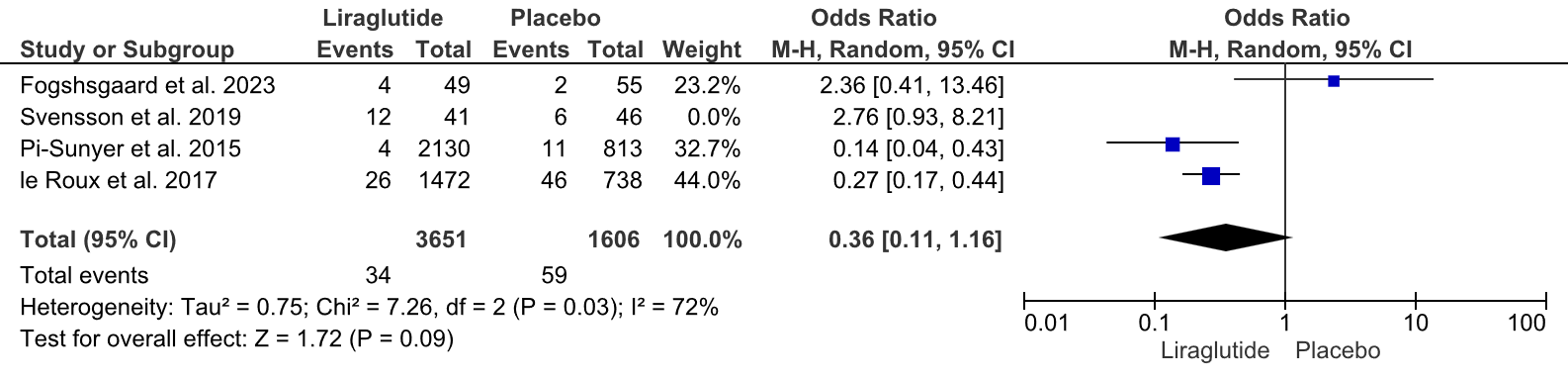
Events, number of participants with Type-2 Diabetes Mellitus; Total, number of participants at risk for Type-2 Diabetes Mellitus; Blue dots, weight of studies; Black blocks, 95% confidence interval of studies; Diamond, estimate with 95% confidence interval
